# Exploiting butyrylcholinesterase inhibitors through a combined 3-D pharmacophore modeling, QSAR, molecular docking, and molecular dynamics investigation†

**DOI:** 10.1039/d3ra00526g

**Published:** 2023-03-23

**Authors:** Sunil Kumar, Amritha Manoharan, Jayalakshmi J, Mohamed A. Abdelgawad, Wael A. Mahdi, Sultan Alshehri, Mohammed M. Ghoneim, Leena K. Pappachen, Subin Mary Zachariah, T. P. Aneesh, Bijo Mathew

**Affiliations:** a Department of Pharmaceutical Chemistry, Amrita School of Pharmacy, Amrita Vishwa Vidyapeetham, AIMS Health Sciences Campus Kochi 682 041 India; b Department of Pharmaceutical Chemistry, College of Pharmacy, Jouf University Sakaka 72341 Saudi Arabia bijomathew@aims.amrita.edu bijovilaventgu@gmail.com aneeshtp@aims.amrita.edu; c Department of Pharmaceutical Organic Chemistry, Faculty of Pharmacy, Beni-Suef University Beni-Suef Egypt; d Department of Pharmaceutics, College of Pharmacy, King Saud University Riyadh 11451 Saudi Arabia; e Department of Pharmacy Practice, College of Pharmacy, AlMaarefa University Ad Diriyah 13713 Saudi Arabia; f Pharmacognosy and Medicinal Plants Department, Faculty of Pharmacy, Al-Azhar University Cairo 11884 Egypt

## Abstract

Alzheimer's disease (AD), a neurodegenerative condition associated with ageing, can occur. AD gradually impairs memory and cognitive function, which leads to abnormal behavior, incapacity, and reliance. By 2050, there will likely be 100 million cases of AD in the world's population. Acetylcholinesterase (AChE) and butyrylcholinesterase (BuChE) inhibition are significant components of AD treatment. This work developed models using the genetic method multiple linear regression, atom-based, field-based, and 3-D pharmacophore modelling. Due to internal and external validation, all of the models have solid statistical (*R*^2^ > 0.81 and *Q*^2^ > 0.77) underpinnings. From a pre-plated CNS library (6055), we discovered a hit compound using virtual screening on a QSAR model. Through molecular docking, additional hit compounds were investigated (XP mode). Finally, a molecular dynamics simulation revealed that the Molecule5093-4BDS complex was stable (100 ns). Finally, the expected ADME properties for the hit compounds (Molecule5093, Molecule1076, Molecule4412, Molecule1053, and Molecule3344) were found. According to the results of our investigation and the prospective hit compounds, BuChE inhibitors may be used as a treatment for AD.

## Abstract 2

Alzheimer's disease (AD), a neurodegenerative condition associated with ageing, can occur.

## Graphical abstract

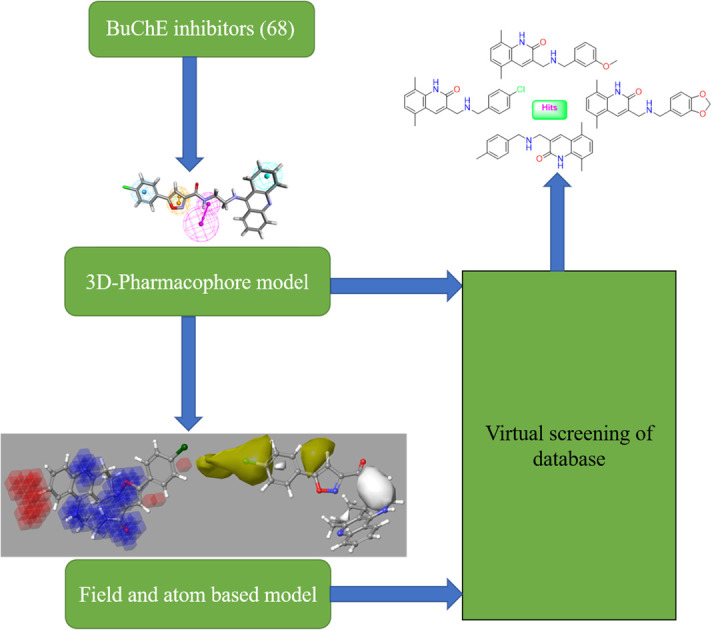

## Introduction

The World Health Organization (WHO) recognises Alzheimer's disease (AD) as a global public health concern.^1^ The most common form of dementia, AD, is a degenerative neurological illness often characterized by mild cognitive impairment and initial memory loss. Numerous factors, such as the extracellular deposition of β-amyloid plaques, the accumulation of intracellular neurofibrillary tangles, oxidative neuronal dysfunction, and inflammatory responses, contribute to the pathogenesis of AD.^2^ The neuromodulators acetylcholine (ACh) and butyrylcholine (BuCh) have been found to be lacking in the brains of AD patients, and inhibiting the major enzymes that hydrolyse ACh and BuCh, respectively, has emerged as an effective therapy option for AD.^3^ Despite being encoded by separate genes on human chromosomes 3(3q26) and 7(7q22), they both share 65% amino acid sequence homology. BuChE has significant pharmacological and toxicological roles because it is highly prevalent in plasma (approximately 3 mg L^−1^) and can break down a wide variety of ester-containing substances. BuChE, for example, can be utilised as a prophylactic scavenger against neurotoxic organophosphates like the nerve gas soman. Prior to now, BuChEs proportional contribution to the control of ACh levels has largely been disregarded, most likely because of its unknown physiological role. Nevertheless, there is mounting proof that both enzymes control ACh levels and may contribute to the onset and progression of AD.^4^

In contrast, BuChE function increases while AChE activity either stabilises or decreases in AD patients' central nervous systems.^5^ The cholinergic shortage assumed to be the leading cause of the deficiencies in cognitive, behavioral, and general functioning that are characteristic of AD can therefore be addressed by targeting these enzymes as potential therapeutic targets.^6^ Since 2003, no brand-new treatments for AD have been approved by the FDA.^7^ BuChE has been linked to AD and reported to have a major pharmaceutical target function. In fact, targeted BuChE inhibition in rats increased ACh levels, enhanced learning, and enhanced long-term potentiation. In a similar manner, *in vivo* BuChE inhibition enhanced learning, memory, and cognitive ability in a mouse model of cholinergic deficit. It is interesting that these tests failed to find any negative effects on peripheral cholinergic function. The reduction of fibrillar A brain plaques in BuChE knockout mice (up to 70%), which suggests that the lower BuChE activity may be helpful in AD, is another interesting observation. Therefore, it is reasonable to expect that the discovery of exceptionally potent and targeted BuChE inhibitors may represent a workable treatment approach for AD.^8–12^

The active subunits of mammalian BuChE generally have 574 amino acid residues.^13^ The 3-D structure of BuChE exhibits the typical/hydrolase fold with a core-sheet and -helices on either side. With two loops surrounding the active site's sides, BuChE's active site gorge is larger than that of AChE and resembles a bowl rather than a deep, narrow canyon. The gorge of BuChE contains around 40% less aromatic residues than the gorge of AChE.^14^ BuChE has a 50–60 diameter and a globular shape. Around halfway down the globule, at a distance of 20 from the surface, the active site is located at the bottom of an opening known as the “active site gorge”.^15–18^ The four subsites that make up the active site gorge are the acylation site, which contains the catalytic residues, the choline-binding pocket, the acyl-binding pocket, and the peripheral anionic site (PAS), which is situated at the rim of the gorge and serves as the first binding site for positively charged substrates and inhibitors. The catalytic triad Ser198, His438 and Glu325 located in the acylation site are responsible for the hydrolytic cleavage of substrates.^14,19,20^ Trp82, a crucial residue solely involved in cation-interactions, is located in the choline-binding pocket. Trp82 is essential for binding positively charged groups of ligands, such as the quaternary ammonium of choline, as shown by site-directed mutagenesis and reactivity labelling.^14,21^ The most notable modification is the acyl-binding pocket, which accepts the acyl moiety of the substrate during catalysis. Leu286 and Val288 replace Phe286 and Val288 in AChE, allowing BuChE to bind and hydrolyze more important ligands and substrates.^22^ Three significant aromatic residues from the PAS of AChE are missing from the rim of the BuChE gorge. As a result, it's frequently thought that BuChE lacks a PAS, at least not one that's comparable to AChEs. Due to the vast cavity of this fissure, BuChEs accept a greater spectrum of substrates and antagonists than AChEs do.^23–25^

Muof evidence points to BuChE as a more beneficial treatment option for moderate-to-severe forms of AD than the more traditional AChE strategy. Because the additional functionalities may significantly enhance the therapeutic effects, patent-protected BuChE antagonists with multifunctional features can open up new therapeutic possibilities.^26^ According to prior research, the most common chemical structures that selectively inhibit BuChE as opposed to AChE include indoline derivatives,^27^ melatonin and its oxidative metabolites,^28^ piperidine analogues,^29^ and substituted benzo-chromone compounds.^30^ The investigation of BuChE inhibitors continues. Because treating AD symptoms by lowering BuChE activity is successful.^31,32^ The medications now used to treat AD symptoms work by restricting synaptic acetylcholine's breakdown. Drug development and research processes benefit greatly from using of computer software's drug design methodologies. The quantitative structure–activity relationship, or QSAR, is one of the efficient and practical approaches to drug design.^33,34^ Understanding the structural characteristics of the inhibitors and target receptors involved in a specific biological process through structure–activity analysis enables the development of more potent inhibitors.^35–37^

The current study, the pharmacophore modelling, field and atom-based QSAR models, and GA-MLR-based models to evaluate the QSAR of a series of 6-methoxy-1-tetralone, 3-aminobenzofuran, arylisoxazoles, quinolotacrine, and methylindolinone-1,2,3-triazole derivatives.^38–42^ In addition, the created models are used to create new BuChE inhibitor models with enhanced bioactivity. Additionally, the developed compounds are subjected to investigations of their affinity for the BuChE enzyme, drug-like properties, and molecular dynamics to determine the stability of their complexes with the target receptor, respectively.

## Material and method

### Dataset for QSAR

The choice of chemical moieties is a crucial step in creating a 3-D QSAR pharmacophore model since it affects the properties of the pharmacophores that are produced. In this study all molecules are taken by same group from Akbarzadeh et, al. in this dataset total 68 compounds for used as a BuChE inhibitors.^38–42^ After starting using BioChemdraw, the inhibitors' 2-D chemical structures. Dataset were curated by using alvamolecule.^43^ Additionally, the Discovery Studio (DS) v2 (https://www.accelrys.com/) programme transformed the structure to 3-D.^44^ The energy of the three-dimensional structure of inhibitors was lowered in DS using the Steepest Descent technique. Training and test sets were made after the residual compounds' biological activity was reduced by four orders of magnitude. The compounds from the training set were used to create the pharmacophore hypothesis, and the results from the test set were used to validate the hypothesis. The reported IC_50_ values for the chosen collection of drugs against BuChE varied from 39 to 100000 nM.

Same dataset structural files must be imported into the OPLS4 force field in maestro (V 13.4) for the macromodel minimization (Schrödinger, LLC, NY, 2022) to be successful.^45^ Additionally, the ligand alignment module is used to align every molecule. The pharmacological and structural properties of that dataset are varied. We utilized the well-known formula pIC_50_ = log(IC_50_ × 10^−9^) to convert the IC_50_ (nM) values to pIC_50_. The BuChE pIC_50_ is the negative logarithm^46^ of the IC_50_ value derived from the BuChE inhibition experiment, expressed in micromolar (μM) or nanomolar (nM). For modelling investigations, all pIC_50_ values were additionally taken into account. The same dataset was used to create GA-MLR (genetic algorithm multiple linear regression) models, which were then internally and externally validated using the well-known programme QSARINS ver. 2.2.2.^47^

### Pharmacophore modeling

Finding new scaffolds is made simple and quick by employing pharmacophore modelling, which may be produced using either ligands or the structure of the target biomolecule. The ligand-based hypothesis can be further refined using the structure–activity relationship of the compounds in the training set or property shared by the most active ligands. In the current investigation, we used information about the biological activity data of BuChE inhibitors that were already known to construct a 3-D pharmacophore model. The DS 3-D Pharmacophore Generation module was used to create quantitative pharmacophores. The essential characteristics in the compounds of the training set were identified using the DS Feature Mapping module.^48^ The information of the feature mapping findings was then used to generate the hypothesis. The uncertainty value, which reflects the ratio of each compound's true biological activity to its measured biological activity, was set at 2.0 prior to hypothesis creation. Other parameters were left as defaults at the same time. The quantitative hypothesis generation used attributes with a minimum and maximum of 0 to 5. The best hypothesis was chosen based on the following criteria: greatest correlation coefficient (*R*^2^), lowest overall cost, fit values, and root mean square deviation among the 10 created models (RMSD).

To support the selected hypothesis, Fischer's randomization test and test set analysis were performed.^49,50^ The statistical applicability of the hypothesis was assessed using Fischer's method. The hypothesis is regarded significant if the total cost is less than the randomly generated hypothesis. The DS Hypogen algorithm creates 9 random spreadsheets with a 90% confidence level for the Fischer's test. The chosen hypothesis was tested using the test set approach to see if it could correctly predict and categorise the chemical compounds based on their range of biological activity. Nine compounds with four orders of magnitude were randomly selected from literature sources to test the idea.

### Field-based and atom-based 3-D QSAR modelling

The phase module of the maestro (V 13.4.132) interface of Schrodinger's utility was used to create 3-D QSAR models. To better comprehend the connection between structural attributes and biological activity, we routinely develop both atom-based and field-based 3-D QSAR models. According to previously defined and widely accepted research guidelines, all of the models were built using the “Phase” module's default parameters and a 70% : 30% random selection of training and test sets.^51–55^ We made sure, nevertheless, that the models we built were not the result of chance, and we further evaluated them for both internal and external validations of their statistical significance. Datasets were further divided into training and test sets, and the varied chemical spaces that molecules adopted were examined. To guarantee high model reliability, both sets of molecules included both active and inactive ones. With the use of a PLS factor of 5, we applied randomization to divide the dataset into 70% training molecules and 30% test molecules for both 3-D QSAR models. Visualizing the molecules from the practice and test sets allowed for a further examination of the software's random selection (diversity among dataset molecules in training and test sets). We have made sure to keep the grid spacing at one for the chosen hypothesis. For the BuChE dataset, we produced 3 models for atom-based 3-D QSAR models and 5 models for field-based models. For the field-based and atom-based models, respectively, we have included 19 and 20 molecules in the test set and 49 and 48 molecules in the training set. Statistical criteria were used to choose the best models. The Gaussian field-based 3-D QSAR models included Gaussian steric, electrostatic, hydrophobic, hydrogen bond donor, and hydrogen bond acceptor components. In field-based models, we considered employing Gaussian intensities as descriptors (as independent variables). The most effective 3-D QSAR models were built to display 3-D contour maps connected to structural elements. QSAR model visualisation is essential for the scaffolds to be tuned more successfully.

### Molecular docking

From the Protein Data Bank (http://www.rcsb.org), we carefully chose and gathered the essential crystal structure of the BuChE (PDB ID: 4BDS, Resolution: 2.10).^55^ We carefully reviewed the literature before choosing the PDB ID. Workflow in maestro is imported with this PDB. The crystal structures were optimized and minimized by removing water molecules, modifying side chain protonation states, and incorporating missing hydrogen using the Protein Preparation Wizard programme. It was further processed for grid creation after the necessary protein had been digested. A receptor grid was subsequently created around the co-crystallized ligand of the enzyme in order to determine the binding location. The compounds were prepared for docking using the LigPrep tool, and the OPLS-2005 force field was employed.^56^

### Molecular dynamic

The Desmond package (Desmond V 7.2) was installed on a Dell Inc. precision 7820 Tower with the configuration Ubuntu 22.04.1 LTS 64-bit, Intel Xenon (R) silver 4210R, and NVIDIA Corporation GP104GL (RTX A 4000) graphics processing unit to perform the MD studies for the lowest docking pose of compound 4eut BX7 1 with 4BDS using the OPLS2005 force field. System box parameters included an orthorhombic box shape and a 10 × 10 × 10 Å^3^ buffer capacity. In addition to 0.15 M NaCl ion concentrations being introduced to the system for neutralization, explicit water molecules (SPC) were also used to prepare the system.^57^ NPT ensembles were used in MD simulations at 310 K with Nose–Hoover temperature coupling and at 1.01 bar constant pressure with Martyna–Tobias–Klein pressure coupling. A RMSD, RMSF, and protein ligand contact analysis across all Cα atoms was built during the 100 ns MD simulation to evaluate the domain correlations. Following the MD run, 1000 frames were generated for each MD trajectory at 100 ps intervals to study the kinetics of protein-ligand interaction.

### QSARINS based MLR models

The MLR-based model employed the same dataset of BuChE inhibitor. Molecular descriptors were lastly computed using PaDEL, Chempoy, and RDkit.^58–60^ The MLR model was created using 2-D and 3-D data by QSARINS version 2.2.2.^47^

## Result and discussion

### 3-D Pharmacophore modeling and validation

Through 45 training sets of chemicals, the 3-D pharmacophore creation module of Accerlys Discovery Studio 2.5 generated with ten hypothesis's (Table 1). Additionally, HypoGen offers two hypothetical expenses (expressed in bit units) to aid in evaluating the reliability of the hypothesis. The first is a fixed cost (cost of an ideal hypothesis), which represents the most straightforward model that accurately predicts all the data, and the second is null cost (cost of null hypothesis), which represents the most expensive pharmacophore with no features and which calculates activity as the average of the activity data of the molecules in the training set. The highest cost difference (188.17), lowest root means square deviation (RMSD = 0.8474), and best correlation coefficient (*r* = 0.911589) made Hypo1 the most significant hypothesis. For Hypo1, the overall cost was 192.958 and the fixed cost and null cost values were 150.617 and 381.131, respectively. This observation was considerably more in line with the fixed cost than the null cost.

**Table:** As a result of the automated HypoGen pharmacophore creation procedure, information of statistical importance and predictive potential is delivered as cost values measured in bits for the top 10 hypothesis

Hypothesis	Total cost	Cost difference	Correlation	RMSD	Features
Hypo1	192.958	188.17	0.911589	1.3717	HBD, Hy, Hy, and RA
Hypo2	211.089	170.04	0.871199	1.63809	HBD HyRA, HyRA, and RA
Hypo3	211.115	170.02	0.871429	1.63679	HBD, Hy, Hy, and RA
Hypo4	212.97	168.16	0.868245	1.65578	HyA, Hy, RA, and RA
Hypo5	214.504	166.63	0.863392	1.68348	HBD, Hy, Hy, and RA
Hypo6	215.268	165.86	0.861502	1.69421	HBD, Hy, RA, and RA
Hypo7	216.183	164.95	0.859302	1.70659	HBA, HyA, Hy, and RA
Hypo8	216.671	164.46	0.859612	1.70501	HBA, HBD, Hy, and RA
Hypo9	216.954	164.18	0.857474	1.71679	HBA, HyA, Hy, and RA
Hypo10	218.827	162.30	0.853609	1.73817	HBD, HyA, RA, and RA

**Fig. 1:**
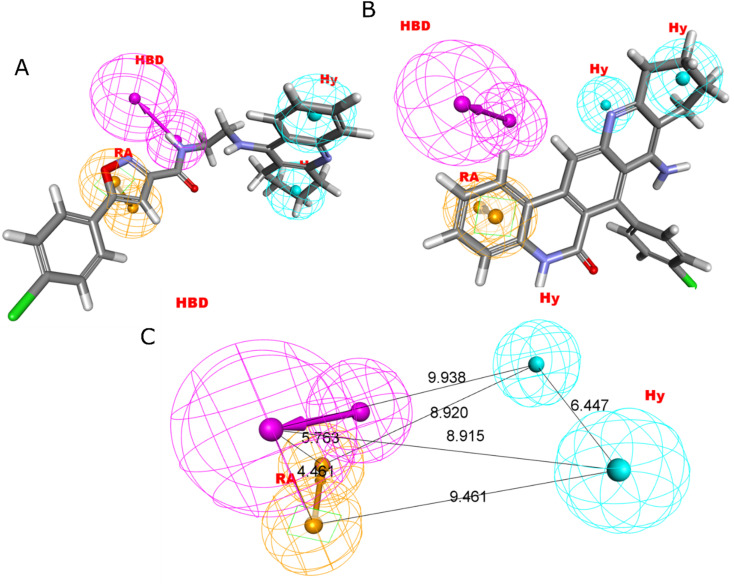
Two hydrophobic (Hy), one hydrogen bond acceptor (HBD), and one ring aromatic (RA) feature are present in Hypo1. (A and B) The most and least active compounds in the training set, as determined by Hypo1's pharmacophore mapping results. (C) The most accurate pharmacophore model, Hypo1, displays chemical characteristics and distance restrictions Å.

Four characteristics make up the best hypothesis (Hypo1): two hydrophobic (Hy), one hydrogen bond donor (HBD), and one aromatic ring characteristic (RA). The best pharmacophore (Hypo1) with compound 1 (IC_50_ = 39 nM) and compound 57 (IC_50_ = 100 000 nM) in the training set, respectively, are depicted in Fig. 1a and b. The distance restrictions in the optimum pharmacophore are shown in Fig. 1c (Hypo1). The mapping difference between the most and least active compounds suggests a potential variation in the inhibitory effects of these compounds against BuChE. The best pharmacophore hypothesis (Hypo1) experimental (log Activ) and estimated (log Estimate) activities for 45 training set chemicals are displayed in (Table 2A).

**Table:** Log experimental and log predicted data values of training set (A) and test set (B) molecules against Hypo1

Code	log Activ	log Estimate	Uncertainty	Error	Fit value
**(A) Training set**					
Compound 1	1.59106	1.82707	2	1.72188	7.17923
Compound 4	1.87506	1.67504	2	−1.58499	7.33126
Compound 5	1.93952	1.89283	2	−1.11349	7.11347
Compound 6	1.94448	1.64845	2	−1.97712	7.35785
Compound 9	2.30535	2.91648	2	4.08436	6.08982
Compound 11	2.61595	2.73943	2	1.32886	6.26687
Compound 12	2.74036	3.96789	2	16.8859	5.03841
Compound 13	2.85491	3.53676	2	4.80668	5.46954
Compound 14	2.91381	3.90159	2	9.7225	5.10471
Compound 15	2.94596	3.52668	2	3.80823	5.47962
Compound 19	3.46909	3.52717	2	1.14311	5.47913
Compound 21	3.70329	4.10685	2	2.53255	4.89945
Compound 22	3.75335	3.66194	2	−1.23429	5.34436
Compound 23	3.75404	3.85791	2	1.27017	5.14839
Compound 26	4.07192	3.67446	2	−2.49724	5.33184
Compound 27	4.1066	4.09167	2	−1.03498	4.91463
Compound 28	4.11025	3.88105	2	−1.69513	5.12525
Compound 30	4.14457	3.94727	2	−1.57509	5.05903
Compound 32	4.2048	3.6727	2	−3.40481	5.3336
Compound 35	4.27346	3.95859	2	−2.06479	5.04771
Compound 36	4.30643	4.84845	2	3.48361	4.15785
Compound 38	4.33925	4.94108	2	3.99786	4.06522
Compound 39	4.35315	3.96434	2	−2.44797	5.04196
Compound 40	4.40364	3.94209	2	−2.89428	5.06421
Compound 41	4.41863	4.60815	2	1.54709	4.39815
Compound 43	4.43965	4.63056	2	1.55206	4.37574
Compound 44	4.45117	4.95774	2	3.21044	4.04856
Compound 45	4.45682	3.82148	2	−4.3186	5.18482
Compound 46	4.49388	3.97527	2	−3.30068	5.03103
Compound 47	4.59693	4.94031	2	2.20489	4.06599
Compound 49	4.75568	4.35761	2	−2.5008	4.64869
Compound 50	4.79246	4.9406	2	1.4065	4.0657
Compound 68	5	4.50557	2	−3.12198	4.50073
Compound 62	5	4.58469	2	−2.60201	4.42161
Compound 54	5	4.67755	2	−2.10112	4.32875
Compound 60	5	4.71317	2	−1.93567	4.29313
Compound 51	5	4.75109	2	−1.77382	4.25521
Compound 63	5	4.76056	2	−1.73558	4.24574
Compound 55	5	4.78102	2	−1.65568	4.22528
Compound 52	5	4.94075	2	−1.14616	4.06555
Compound 66	5	4.95207	2	−1.11669	4.05423
Compound 67	5	4.9873	2	−1.02968	4.019
Compound 64	5	5.01155	2	1.02695	3.99475
Compound 65	5	5.01165	2	1.02718	3.99465
Compound 57	5	5.02938	2	1.07	3.97692

**(B) Test set**					
Compound 56	5	3.40073	2	−39.7436	5.60557
Compound 59	5	5.00774	2	1.01799	3.99856
Compound 53	5	4.94032	2	−1.1473	4.06598
Compound 61	5	5.03042	2	1.07256	3.97588
Compound 58	5	5.03856	2	1.09284	3.96774
Compound 48	4.62189	4.46493	2	−1.43537	4.54137
Compound 42	4.43329	4.94096	2	3.21864	4.06534
Compound 37	4.31361	3.51964	2	−6.22263	5.48666
Compound 34	4.26529	3.67355	2	−3.90608	5.33275
Compound 33	4.2393	3.47698	2	−5.78528	5.52932
Compound 31	4.20137	3.50781	2	−4.93815	5.49849
Compound 29	4.13799	4.72925	2	3.90181	4.27705
Compound 25	4.02284	3.49931	2	−3.33837	5.50699
Compound 24	3.79782	3.32285	2	−2.98516	5.68345
Compound 20	3.67943	4.92702	2	17.6845	4.07928
Compound 18	3.4609	4.20281	2	5.51962	4.80349
Compound 17	3.02694	2.15063	2	−7.52161	6.85567
Compound 16	2.99564	3.46244	2	2.92955	5.54386
Compound 10	2.57054	1.91333	2	−4.54164	7.09297
Compound 8	2.01703	2.41309	2	2.48915	6.59321
Compound 7	1.94448	2.18456	2	1.7381	6.82174
Compound 3	1.70757	2.63075	2	8.3788	6.37555
Compound 2	1.6721	1.82876	2	1.43436	7.17754

**Fig. 2:**
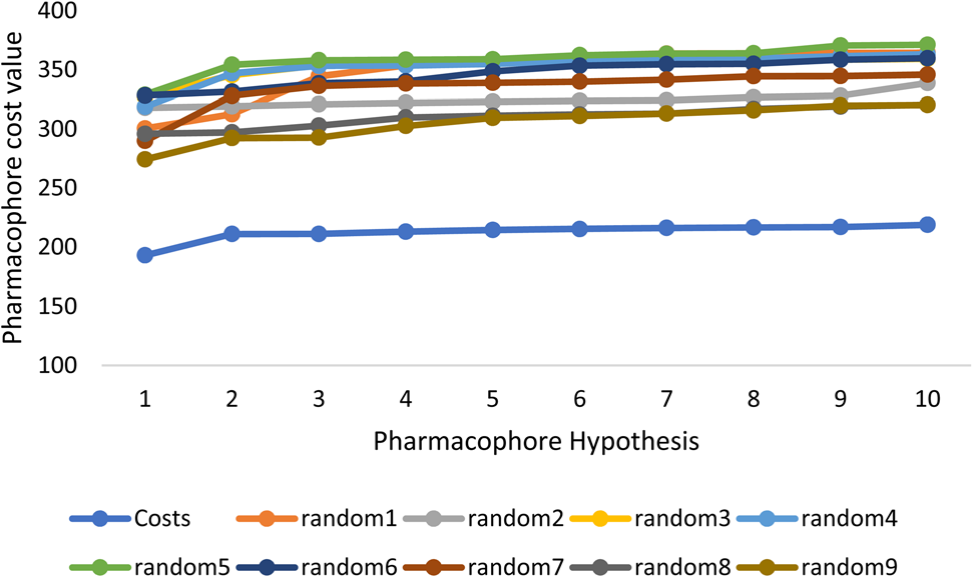
The cost difference between random runs and HypoGen. The chosen level of confidence was 90%.

**Fig. 3:**
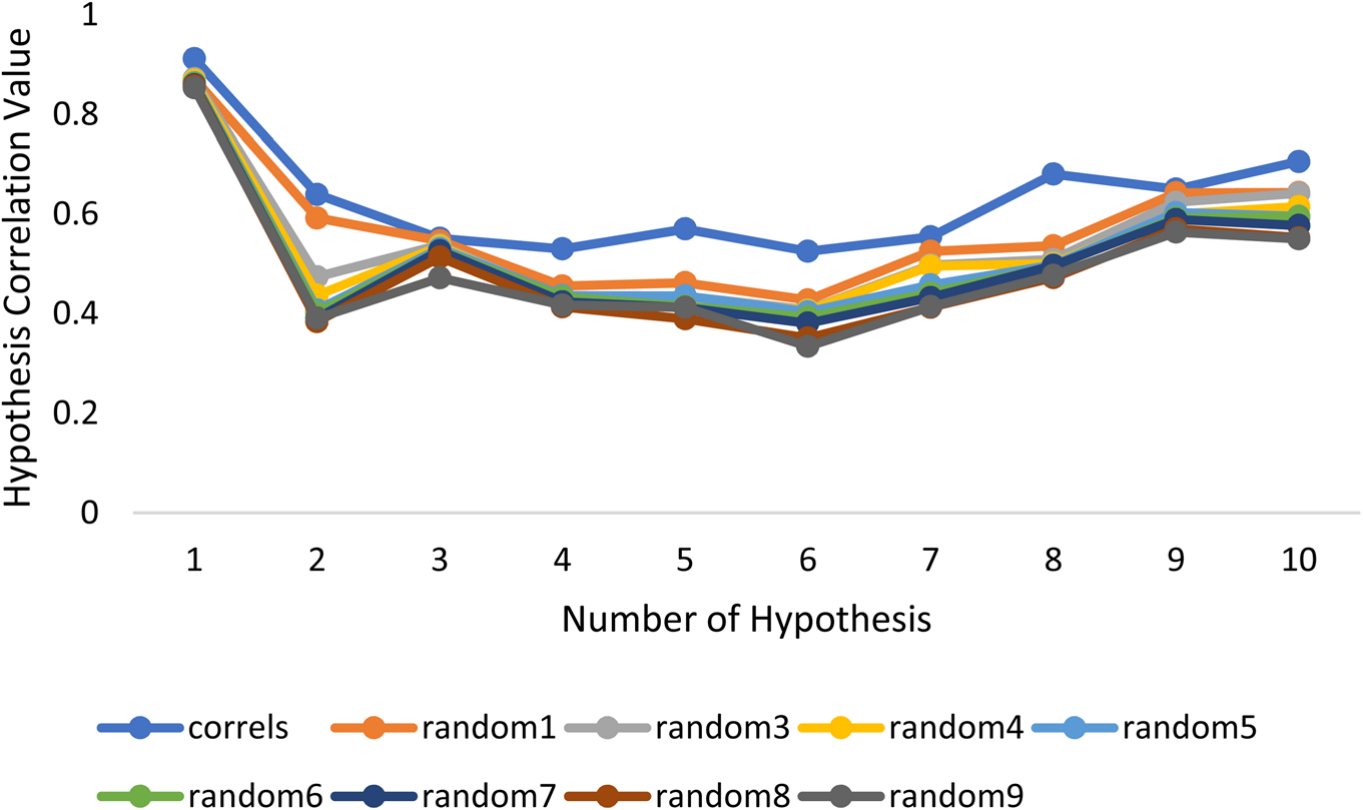
The deviation in correlation between HypoGen and scrambled runs. The chosen level of confidence was 90%.

**Fig. 4:**
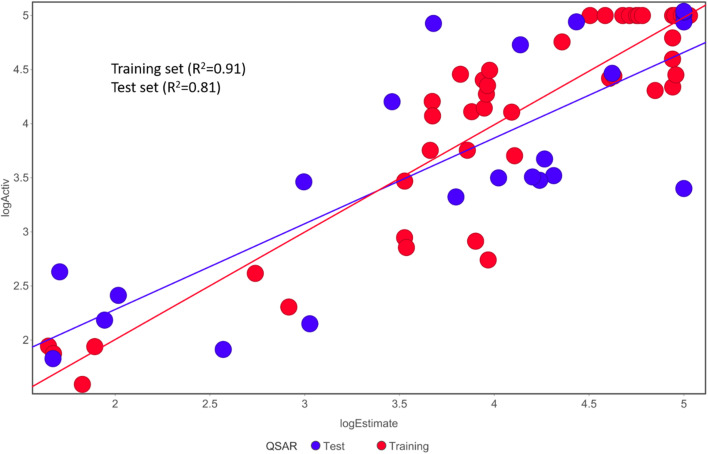
The graph shows the relationship between the experimentally determined activities of the substances in the training set and test set and those predicted by Hypo1.

Fischer's randomization test (FRT) and test set validation were employed to assess the representative hypothesis. Using the genuine biological activity values at the 90% confidence level, Fischer's validation technique constructed nine randomly chosen spreadsheets of the compounds from the training set to test the statistical significance of the hypothesis. Because Hypo1 was the least expensive of the nine randomly generated hypotheses in Fig. 2, it can be concluded that it was not produced by accident. Additionally, the connection between Hypo1 and the randomly produced hypothesis was assessed, and it was discovered that Hypo1 had the highest correlation among the set of randomly formed hypotheses (Fig. 3). The test set strategy is used to establish if the pharmacophore model can forecast the actions of the test set series' extraneous compounds. The test includes 23 molecules. The inhibitory activity of the test set's compounds ranged from 47 to 100 M. For 23 test set chemicals, the optimal pharmacophore hypothesis (Hypo1) experimental (log Activ) and estimated (log Estimate) activities are shown in (Table 2B). Furthermore, Hypo1 demonstrates a significant correlation between the predicted and actual biological activity in the training set (*R*^2^ = 0.91) and test set (*R*^2^ = 0.8) (Fig. 4). The results from the Hypo1 validation demonstrate that the selected hypothesis met the criteria for the best pharmacophore model for virtual screening of druglike datasets, as suggested by prior research.

### Atom-based and field-based 3-D QSAR models

We constructed atom- and field-based 3-D QSAR models and tested their reliability using both internal and external validation criteria. The 3-D QSAR models' robustness, stability, and prediction abilities were examined using the leave-one-out (LOO) cross-validation method. We analyse both models for 20 test chemicals for the atom-based and field-based 3-D QSAR models (Table S1†). The PLS factors used in the construction of the field-based and atom-based QSAR models are 5 and 3, respectively (Tables 4 and 5). The internal validation parameters for both atoms-based and field-based 3-D QSAR models are provided in Tables 3 and 4, respectively. Both models were evaluated based on the outcomes after passing predetermined criteria and gaining external validation. Finally, we found that the 3-D QSAR models we developed had a very high statistical significance.

**Table:** PLS parameter for field based and atom-based QSAR statics for BuChE, selected PLS

PLS	SD	*R* ^2^	*R* ^2^ CV	*R* ^2^ Scramble	Stability	*F*	*P*	RMSE	*Q* ^2^	Pearson-*r*
**(A) Field based QSAR**										
5	0.4481	0.8463	0.7436	0.4009	0.971	46.3	5.15 × 10^−16^	0.44	0.7973	0.8944

**(B) Atom based QSAR**										
3	0.4763	0.8173	0.7187	0.293	0.983	65.6	2.80 × 10^−16^	0.47	0.7784	0.9184

The field based and atom-based 3-D QSAR statistics for atom type fraction for BuChE, selected PLS

**Table:** 

(A) Field based QSAR					
PLS	Gaussian steric	Gaussian electrostatic	Gaussian hydrophobic	Gaussian H-bond acceptor	Gaussian H-bond donor
5	0.396	0.094	0.231	0.166	0.114

**Table:** 

(B) Atom based QSAR			
PLS	H-bond donor	Hydrophobic/non-polar	Electron-withdrawing
3	0.048	0.681	0.247

As evidenced by *Q*^2^ > 0.5, *R*^2^ train > 0.6, *R*^2^ test > 0.6, *r*20 *r*02′ 0.3, 0.85 *k* > 1.15, 0.85 *k*′ > 1.15, (*r*2 − *r*20)/*r*2 > 0.1, and (*r*2 *r*′0 2)/*r*2 being all significant values below the threshold, the chosen model's modelling statistical parameters met the Golbraikh and Tropsha acceptance conditions. The regression equation's “*r*2” coefficient indicates how much of the overall variation in the dependent variables it can account for. A QSAR model is considered to be in good shape when its high *r*2 values fall between 0.6 and 0.9. We found reduced RMSE values of 0.44 and 0.44 for the atom-based and field-based 3-D QSAR models, respectively. It is clearly accepted that a low RMSE value indicates more accuracy because the observed and simulated data are closely connected. A lower RMSE hence results in greater model performance. The departure from the regression line is also known as the standard deviation (SD). This is a gauge of how accurately the function produced from the QSAR analysis predicts the biological activity that has been observed. The QSAR is better the lower the SD value. We created a 3-D QSAR model for BuChE that kept the best model and had lower SD values (SD for field-based 3-D QSAR: 0.4481; SD for atom-based 3-D QSAR: 0.4763). The Fisher statistic can be used to evaluate the regression model's statistical significance (*F*). For a set of degrees of freedom (*p*), where *p* is the number of model descriptors and *n* is the number of molecules, the variance ratio, also known as the *F*-value, is a measurement of how much variation is explained in comparison to how little is explained. We selected atom-based and field-based 3-D QSAR as the best model for the BuChE datasets; the *F* value is displayed in the tables.

**Fig. 5:**
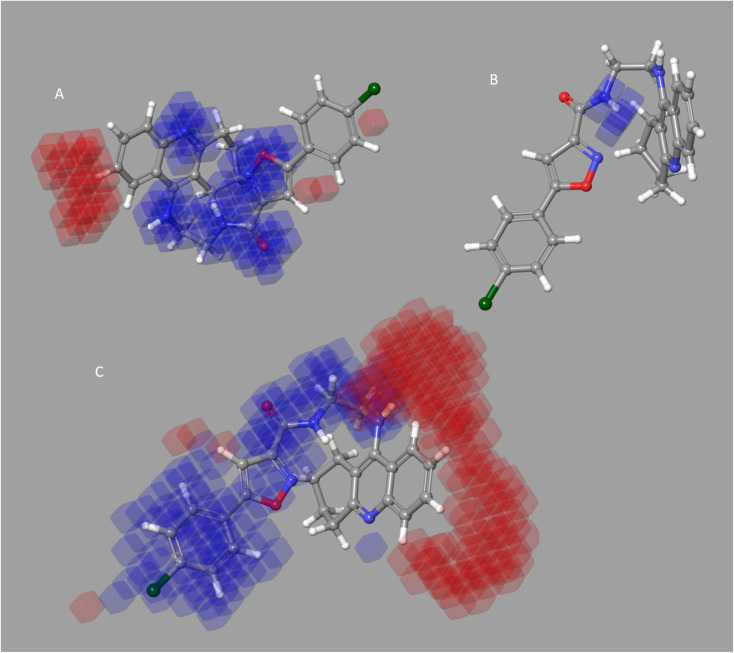
BuChE for a 3-D atom-based contours map with the most active molecule, blue biologically favorable regions, and red biologically unfavorable regions. (A) Withdrawing electrons; (B) donor of H-bonds; and (C) hydrophobic.

It seems to reason that the structural traits of the core moiety that causes activity, like occlusion maps, may be strongly tied to biological activities. While blue occlusion maps/contours represented increased biological activity, red occlusion maps/cubes indicated a decline in biological activity. We selected representative compound 1 from the BuChE datasets for the best QSAR occlusion map presentation. The 1,2,3,4-tetrahydroacridine in the blue area moiety and the isoxazole and to connected linker showed a favorable site for electron withdrawing groups (EWG) on the occlusion maps (Fig. 5A). The linker between isoxazole and 1,2,3,4-tetrahydroacridine in Fig. 5B's positive ion occlusion maps display the H-bond donor's preferred area. The hydrophobic area, phenyl, and isoxazole moiety, along with the connected linker, are shown in Fig. 5C as being advantageous for bioactivity. The contours associated with additional substitutions are shown in Fig. 5. Table 5B contain the PLS parameters and atom type percentages; the majority of the factor depends on the hydrophobic and electro withdrawing groups.

**Table:** Selected model 1 for statistical validations parameters^a^

Statistical parameter	Model-1
**Fitting**	
*R*_tr_^2^	**0.9083**
*R*_adj_^2^	0.8949
*R*_tr_^2^ − *R*_adj_^2^	0.0134
LOF	0.1909
*K*xx	0.3471
Δ*K*	0.0728
RMSE_tr_	0.3277
MAE_tr_	0.2599
RSS_tr_	5.1553
CCC_tr_	0.9520
*s*	0.3546
*F*	67.7200

**Internal validation**	
*R*_cv_^2^(*Q*_loo_^2^)	**0.8729**
*R* ^2^ − *R*_cv_^2^	0.0355
RMSE_cv_	0.3859
MAE_cv_	0.3065
PRESS_cv_	7.1494
CCC_cv_	0.9338
*Q*_LMO_^2^	0.8634
*R*_Yscr_^2^	0.1289
*Q*_Yscr_^2^	−0.2023

**External validation**	
RMSE_ex_	0.4908
MAE_ex_	0.3937
PRESS_ext_	4.8169
*R*_ex_^2^	0.7442
*Q* ^2^ − *F*^1^	0.7445
*Q* ^2^ − *F*^2^	0.7088
*Q* ^2^ − *F*^3^	0.7945
CCC_ex_	0.8476
**Calc. external data regr. angle from diagonal**	−6.0065°
*R* ^2^-ExPy (predictions by LOO)	0.8735
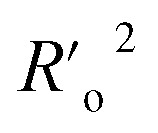	0.8624
*k*′	**0.9954**
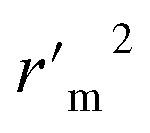	0.7815
*R*_o_^2^	0.8729
*k*	**0.9992**
*r*_m_^2^	0.8522

a A GA-MLR based QSAR model's statistical quality and strength were assessed using the following criteria: (a) internal validation using the leave-one-out (LOO) and leave-many-out (LMO) procedure; (b) external validation; (c) Y-randomization (or Y-scrambling); and (d) satisfying the corresponding threshold value for the statistical parameters: *R*_tr_^2^ ≥ 0.6, *Q*_loo_^2^ ≥ 0.5, *Q*_LMO_^2^ ≥ 0.6, *R*^2^ > *Q*^2^, *R*_ex_^2^ ≥ 0.6, RMSE_tr_ < RMSE_cv_, Δ*K* ≥ 0.05, CCC ≥ 0.80, *r*_m_^2^ ≥ 0.6, (1 − *r*^2^/*r*_o_^2^) < 0.1, 0.9 ≤ *k* ≤ 1.1 or 
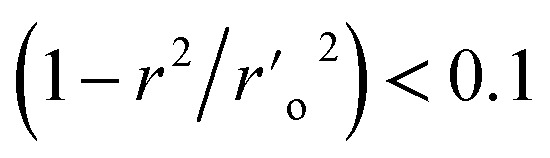
, 0.9 ≤ *k*′ ≤ 1.1,
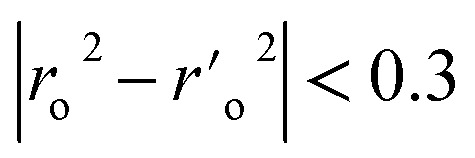
 with RMSE and MAE close to zero.

**Fig. 6:**
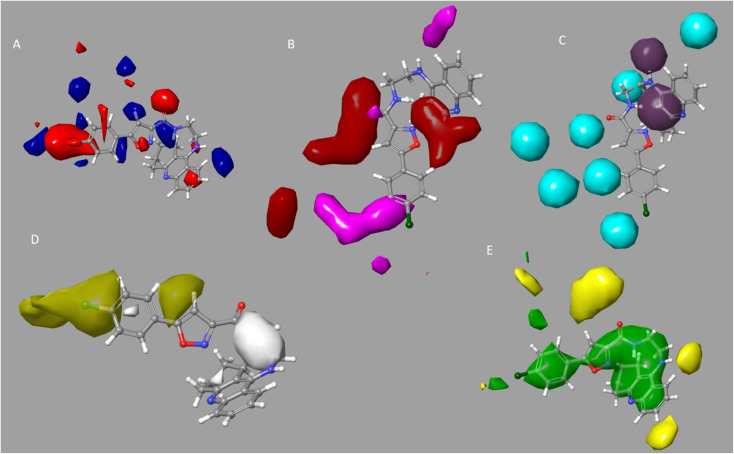
BuChE for a 3-D field-based system with an active molecule contour map. (A) Gaussian electrostatic; the blue region is preferred while the red region is disfavored. (B) Gaussian H-bond acceptor, preferred region is red; magenta is disfavored. (C) Gaussian H-bond donor, where purple is preferred and cyan is not. (D) Gaussian hydrophobic; the yellow region is preferred while the white region is disfavored. (E) Gaussian steric, area where yellow is unfavorable and green is favored.

For the examination of the contour maps made for the field-based 3-D QSAR, we selected active molecules with mixed region for contour maps of the Gaussian electrostatic field (Fig. 6A). Around the 1,2,3,4-tetrahydroacridine moiety, the Gaussian Hydrogen Bond Acceptor Field Contour (Fig. 6B) exhibited favorable (red) areas for BuChE studies. Magenta occlusion maps are not preferred in the vicinity of the phenyl moiety. Around the 1,2,3,4-tetrahydroacridine moiety and linker NH is a preferred region (purple), while away from the active molecule is a disfavored region, according to Gaussian hydrogen bond donor occlusion maps (Fig. 6C) (cyan). In Fig. 6D, the favorable regions (yellow) for BuChE are shown around the phenyl and isoxazole moiety, whereas the disfavored portions (white) are shown for 1,2,3,4-tetrahydroacridine. Maps of the Gaussian steric field occlusion (Fig. 6E) For BuChE, the entire molecule is indicated as favourable (green), whereas the area outside of the isoxazole ring is disfavored (yellow). The field fractions in Table 5A for the developed field-based 3-D QSAR models make it evident that the majority of biological activity depends on Gaussian steric and hydrophobic area.

#### Molecular docking

**Fig. 7:**
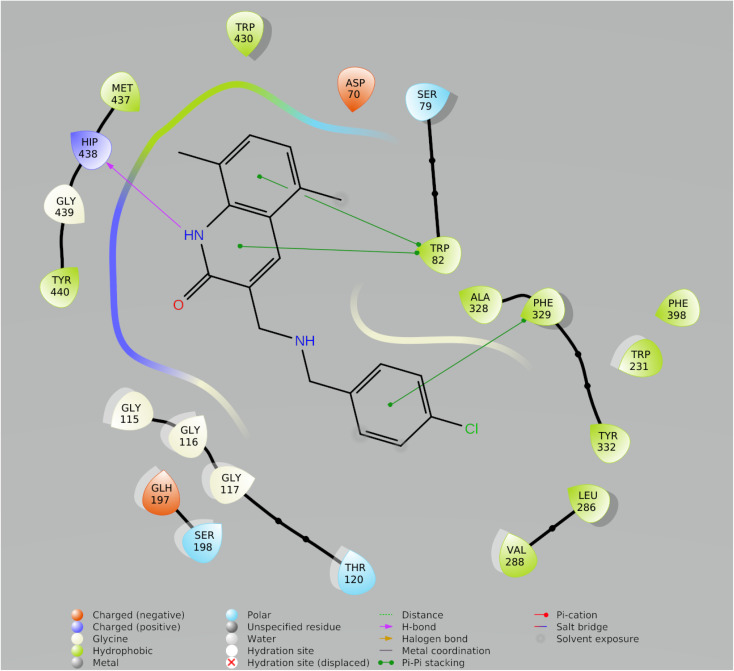
2-D ligand interaction with best Compound Molecule5093 from preplated CNS database.

With the aid of the pre-plated CNS diversity library (6055) (https://www.asinex.com), we performed virtual screening using the Hypo1 (3-D pharmacophore modelling) and PLS 5 (3-D Field based QSAR) models. The hit compound (above fit value = 6.00) from the screening was further assessed using molecular docking. We docked the successful compounds from the virtual screened into the binding cavity of protein 4BDS using the Glide module. We docked molecules using extra precision (XP). The docking scores for the 77 hits, which ranged from −1.86 to −10.49 kcal mol^−1^ in XP mode, were also examined. The top docked hit compounds were Molecule5093, Molecule1076, and Molecule4412, and Molecule1053, with docking scores of −9.94 kcal mol^−1^ (XP mode), −9.85 (XP mode), −9.72 (XP mode), and −9.57 (XP mode), respectively (Table S2†). The binding affinity of these four compounds is greater than that of tacrine (XP = −6.215). The chemical Molecule5093 interacted with significant amino acids in the binding areas of 4BDS. These contained the hydrophobic interactions Tyr440, Met437, Phe329 and Trp82 (double pi–pi stacking), Phe329 with pi–pi stacking, and Hip438 amino acid residues with NH (hydrogen bond). Fig. 6 displays the 2D interaction diagram for the top dock molecules (Fig. 7).

### Molecular dynamic simulation

**Fig. 8:**
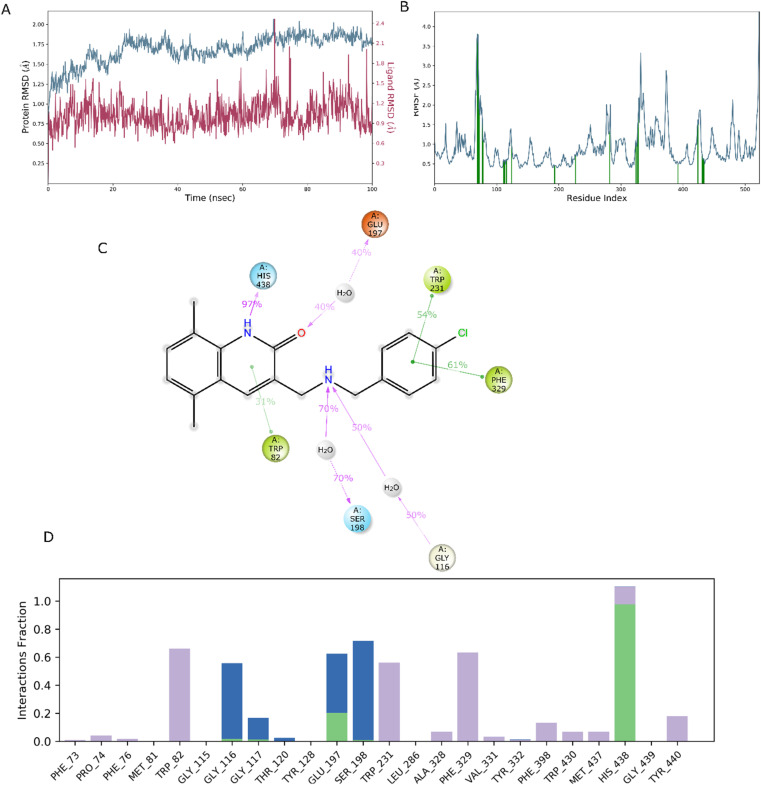
MD simulation, analyze the Molecule5093-4BDS complex. (A) RMSD (Molecule5093 RMSD is presented in red color and protein RMSD is indicated in blue). (B) Individual RMSF for proteins amino acids. (C) Diagram of 2-D interaction. (D) Examining the protein–ligand interactions along the MD trajectory.

In drug discovery research, the MD simulation is utilised to reproduce the nearly accurate or realistic dynamic behaviour of a protein–ligand complex while providing time-affordable grasp of energetic information regarding protein and ligand interactions. This study used MD modelling in biological contexts to simulate Molecule5093 at the 4BDS protein's binding site. The MD trajectories were used to calculate the root-mean-square deviation (RMSD), root-mean-square fluctuation (RMSF), and protein–ligand interactions. Several MD trajectory data analysis for the Molecule5093-4BDS complex are shown in Fig. 8(A–D). Both complexes have been simulated using water molecules. The RMSD figure (Fig. 8A) showed a stable protein–ligand combination over the simulation period, with RMSD values for protein C atoms in the complex with ligand ranging from 0.75 Å to 2.0 Å. The RMSD of the ligand ranged from 0.65 Å to 2.46 Å. With the exception of a little change, the simulation's RMSD for Molecule5093 was found to be constant. At 70 ns, with an RMSD of 2.46, the highest ligand RMSD was determined. Maximum RMSD was measured for protein at 70 ns, when the RMSD value was 2.07 Å. The RMSD plot's overall result demonstrates that the ligand is stable with regard to the protein and its binding site. By computing the RMSF of each protein amino acid residue, the simulation also evaluated the adaptability of the protein system. A high RMSF value suggests a flexible region, whereas a low RMSF value demonstrates the rigidity of the amino acids. The RMSF plot (Fig. 8B) clearly shows that the fluctuations in the amino acids 70–75 and 330–337 have the highest fluctuations. Following major interaction ligand and amino acid, His438 (0.60 Å), Trp82 (1.08 Å), Ser198 (0.46 Å), Gly116 (0.57 Å), Glu197 (0.45 Å), Trp231 (0.73 Å), and Phe329 (0.93 Å). All these residue interacted have RMSF value in the range of 0.45 to 1.08 Å. Hydrogen bonds, hydrophobic, ionic, and water bridges are the three main types of protein ligand interaction. Hydrogen bonds, water bridges, and hydrophobic stability in ligand protein complexes are shown in Fig. 8C and D. It is obvious that Gly116, Ser198, Glu197 and His438 participate in hydrogen bonds to the extent of 50%, 70%, 40%, and 97%, respectively, in 2-D interaction. Trp82, Trp231, and Phe329 with pi–pi stacking participate 31%, 54%, and 61% simulation time. In molecular dynamics simulations, the physiological environment is more accurately mirrored, which will help in understanding binding patterns. The trajectory study and overall MD simulation indicate that the hit compound will inhibit BuChE.

### SwissADME prediction

**Fig. 9:**
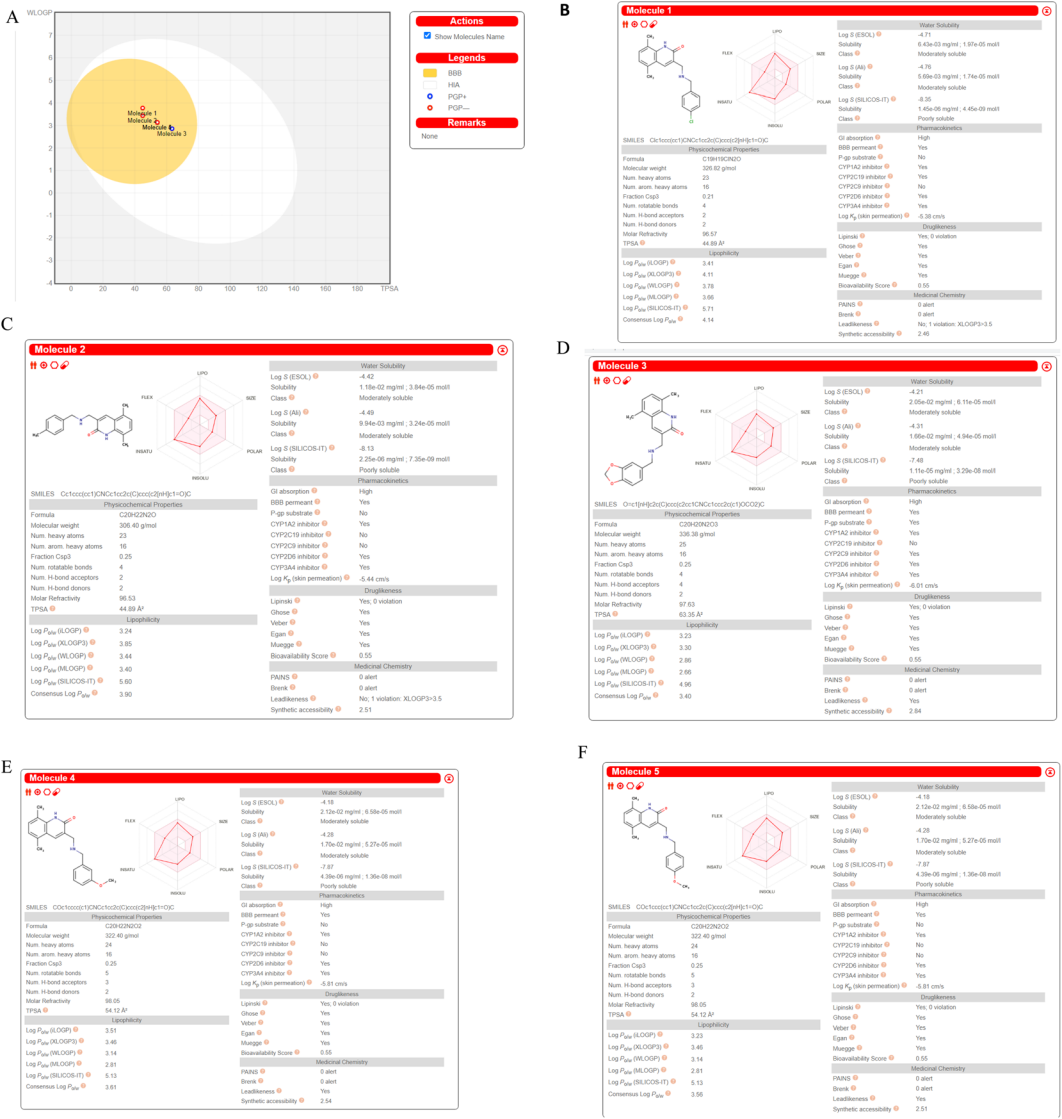
ADME prediction by SWISS ADME (A). Boiled egg (B). Molecule5093 (C). Molecule1076 (D). Molecule4412 (E). Molecule1053 (F). Molecule3344.

We computed predictions of ADME attributes hit compounds (pre plated CNS diverse database). The well-known Lipinski's rule, Ghose, Veber, or Egan rules were not broken by any of the molecules tested for drug similarity.^61^ The overall bioavailability score was determined to be 0.55, while the synthetic accessibility scores for Molecule5093, Molecule1076, Molecule4412, Molecule1053, and Molecule3344 were 2.46, 2.51, 2.84, 2.54 and 2.51, respectively. It (Fig. 9) may have a chance to breach the blood–brain barrier because it falls in the yellow region of the boiled egg model^62^ (BBB). All the hit molecules are GI absorption. It was shown that the hit molecules CYP1A2, CYP2D6, and CYP3A4 are inhibitors, with the exceptions of molecules 5093 for CYP2C9, 1076 for CYP2C9 and CYP2C19, 4412 for CYP2C19, 1053 for CYP2C9 and CYP2C19, and 3344 for CYP2C9 and CYP2C19. All molecules are not P-gp substrates excepts Molecule4412. Hit molecules shown in Fig. 8 ADME property.

### QSARINS based MLR models

The top-ranked model with the most statistical significance was identified, and its ramifications were further assessed, using calculations for both internal and external validations. The following MLR equation serves as a representation of the produced model-1:

1 pIC_50_ = −78.9536 − 0.0452 × RDF155 × 10^23^.4623 × SpMax2_Bhe + 0.2815 × MAXDP2 − 0.0827 × ETA_Beta_ns − 147.6785 × ETA_EtaP_B-4.4339 × bcutp13

#### Multivariate models

##### Model 1 (70% training: 30% test set, 6 parametric)

Current work on the parametric QSARINS 6 model is underway. The Radial Distribution Function 155e (RDF155e) is a representation of the function weighted by the relative Sanderson electronegativities (RDF descriptor). This descriptor and the activity are connected negatively. Largest absolute eigenvalue of the Burden modified matrix – *n* 2/weighted by relative Sanderson electronegativities is what makes up the SpMax2_Bhe representation of the Burden Modified Eigen values descriptor. This description and the activity have a beneficial relationship. Electrotopological State Atom Type Descriptor, or MAXDP2, is a representation of the maximum positive intrinsic state difference in the molecule. Molecular descriptor modelling and prediction of non-ionic organic pesticide soil sorption coefficients. 41, 763–777, Chemosphere There is a good correlation between this description and the bioactivity. The Extended Topochemical Atom descriptor is described by the acronym ETA_Beta_ns. This is represented by the molecule's A measure of electron-richness, which has a bad correlation with activity. The branching index EtaB in relation to molecular size was represented by the Extended Topochemical Atom descriptor ETA_EtaP_B. Negative correlation between this description and bioactivity. A Burden description based on polarizability is called bcutp13. The connection between this descriptor and activity is adverse.

**Fig. 10:**
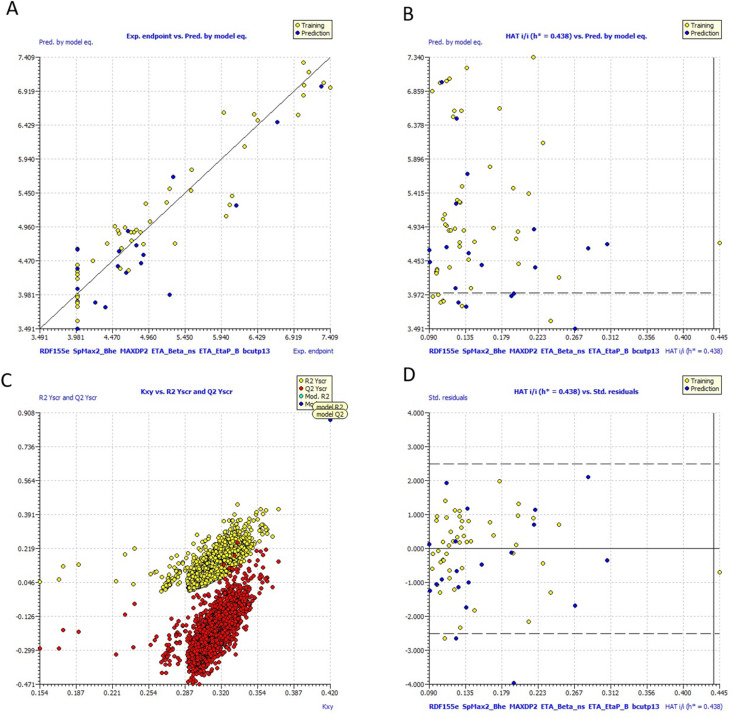
(a) Graph of experimental *vs.* Predicted pIC_50_ values for model 1 (b) Insubria plot for model 1; (c) Y-scrambling plot for model (d)William's plot for model 1.

Fig. 10 includes graphs of experimental *vs.* projected pIC_50_ values, the Insubria plot, the Y-scrambling plot, the William's plot, and the Insubria plot. Information about the whole statistical analysis is also included (Fig. 10). Additional proof for the GA-MLR QSAR model's statistical robustness was supplied by its various cross-validation qualities (*R*^2^cv, RMSEcv, MAEcv, CCCcv, and *Q*^2^ LMO). Greater results for the Tropsha and Golbraikh criterion,^63,64^*Q*^2^*F*^1^, *Q*^2^*F*^2^, CCCex, and *Q*^2^*F*^3^ demonstrated the external predictive power of the suggested models 1. The statistical parameters *R*^2^ = 0.9083 and *Q*_loo_^2^ = 0.8729 are mentioned in Table 5, and the model is deemed to be reliable. All other statistical parameters were found to be within acceptable ranges (additional Tables S3 and S4†).

It would be able to determine the causes of variations in the BuChE inhibitors by creating QSAR models using a range of chemical descriptors.

More descriptor computation data, precise modelling, and less statistical artefacts might result in the creation of improved models, even though the existing QSAR models have several drawbacks.

As a consequence, each of the models developed here shows how all selected chemical features may be integrated, and it also predicts future pIC_50_ values for the aforementioned analogues.

## Conclusion

In summary, we have developed statistically sound 3-D pharmacophore modelling, GA-MLR, atom-based, and field-based 3-D QSAR models for BuChE inhibitors with robust training set, *R*^2^ > 0.81, and test set, *Q*^2^ > 0.77 parameters. We discovered hits compounds using a ligand-based virtual screening method. Additionally, molecular docking (XP mode) was carried out, and we obtained 77 hits. Additionally, we performed a dynamic simulation on the top docking score molecule and discovered that both protein–ligand complexes are stable. We came to the conclusion that the majority of the hit compounds are CYP family inhibitors, BBB permeable, show high GI absorption, and do not violate the Lipinski rule based on Swiss ADME prediction. Overall, the findings of this research will help develop new families of BuChE enzyme inhibitors for the treatment of Alzheimer's illnesses.

## Conflicts of interest

There is no conflict of interest.

## Supplementary Material

RA-013-D3RA00526G-s001
