# Correction: Exploiting butyrylcholinesterase inhibitors through a combined 3-D pharmacophore modeling, QSAR, molecular docking, and molecular dynamics investigation

**DOI:** 10.1039/d3ra90030d

**Published:** 2023-04-06

**Authors:** Sunil Kumar, Amritha Manoharan, Jayalakshmi J, Mohamed A. Abdelgawad, Wael A. Mahdi, Sultan Alshehri, Mohammed M. Ghoneim, Leena K. Pappachen, Subin Mary Zachariah, T. P. Aneesh, Bijo Mathew

**Affiliations:** a Department of Pharmaceutical Chemistry, Amrita School of Pharmacy, Amrita Vishwa Vidyapeetham AIMS Health Sciences Campus Kochi 682 041 India bijomathew@aims.amrita.edu bijovilaventgu@gmail.com aneeshtp@aims.amrita.edu; b Department of Pharmaceutical Chemistry, College of Pharmacy, Jouf University Sakaka 72341 Saudi Arabia; c Department of Pharmaceutical Organic Chemistry, Faculty of Pharmacy, Beni-Suef University Beni-Suef Egypt; d Department of Pharmaceutics, College of Pharmacy, King Saud University Riyadh 11451 Saudi Arabia; e Department of Pharmacy Practice, College of Pharmacy, AlMaarefa University Ad Diriyah 13713 Saudi Arabia; f Pharmacognosy and Medicinal Plants Department, Faculty of Pharmacy, Al-Azhar University Cairo 11884 Egypt

## Abstract

Correction for ‘Exploiting butyrylcholinesterase inhibitors through a combined 3-D pharmacophore modeling, QSAR, molecular docking, and molecular dynamics investigation’ by Sunil Kumar *et al.*, *RSC Adv.*, 2023, **13**, 9513–9529, https://doi.org/10.1039/D3RA00526G.

The authors regret that the email addresses were associated with the incorrect institution. The corrected association is as shown above.

The Royal Society of Chemistry apologises for these errors and any consequent inconvenience to authors and readers.

## Supplementary Material

