# Supplementary material for: Exploiting butyrylcholinesterase inhibitors through a combined 3-D pharmacophore modeling, QSAR, molecular docking, and molecular dynamics investigation
Source: RSC Adv. 2023 Mar 23;13(14):9513–29. doi: 10.1039/d3ra00526g (PMC10035067; doi:10.1039/d3ra00526g)
Supplement: RA-013-D3RA00526G-s001 [file RA-013-D3RA00526G-s001.pdf]

Electronic Supplementary Material (ESI) for RSC Advances.
This journal is © The Royal Society of Chemistry 2023

**Exploiting Butyrylcholinesterase inhibitors through a combined 3-D pharmacophore modeling, QSAR, molecular docking, and molecular dynamics investigation**

Sunil Kumar[a], Amritha Manoharan[a], Jayalakshmi J[a], Mohamed A. Abdelgawad[b,c] , Wael A. Mahdi[d], Sultan Alshehri[d], Mohammed M. Ghoneim[e,f], Leena K Pappachen[a], Subin Mary Zachariah[a], T.P. Aneesh[8*], Bijo Mathew[a*]

[a] Department of Pharmaceutical Chemistry, Amrita School of Pharmacy, Amrita Vishwa Vidyapeetham, AIMS Health Sciences Campus, Kochi 682 041, India.

[b] Department of pharmaceutical chemistry, College of pharmacy , Jouf university, Sakaka 72341, Saudi Arabia.

[c]Department of Pharmaceutical Organic Chemistry, Faculty of Pharmacy, Beni-Suef University, Beni-Suef, Egypt

[d]Department of Pharmaceutics, College of departmenting Saud University, Riyadh 11451, Saudi Arabia.

[e]Department of Pharmacy Practice, College of Pharmacy, AlMaarefa University, Ad Diriyah 13713, Saudi Arabia.

[f]Pharmacognosy and Medicinal Plants Department, Faculty of Pharmacy, Al-Azhar University, Cairo 11884, Egypt

[*]Corresponding Authors:

Bijo Mathew (B. Mathew) (bijomathew@aims.amrita.edu) (bijovilaventgu@gmail.com)

T. P. Aneesh (aneeshtp@aims.amrita.edu)

**Table S1**. BuChE for QSAR predicted and Actual activity

| Ligand Name | QSAR Set | Actual Activity ($pIC_{50}$) | Field-based QSAR for Predicted Activity (PLS 5) | QSAR Set | Atom based QSAR for Predicted Activity (PLS 3) |
|---|---|---|---|---|---|
| 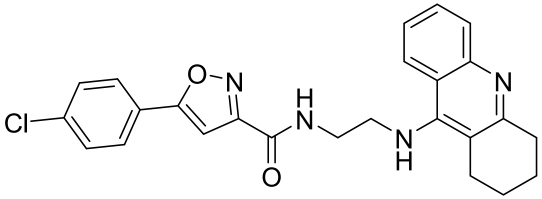 Compound 1 | training | 7.409 | 7.01747 | training | 7.1583 |
| 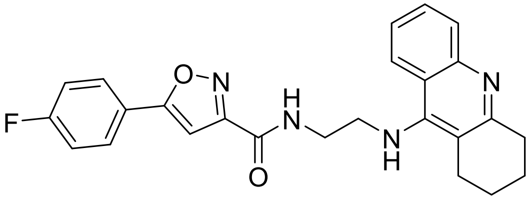 Compound 2 | training | 7.328 | 6.92972 | training | 7.13788 |

| | | | | | |
|---|---|---|---|---|---|
| 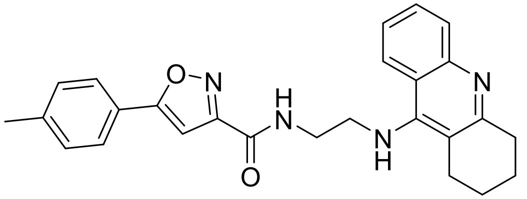 Compound 3 | Test | 7.292 | 6.95671 | training | 7.16023 |
| 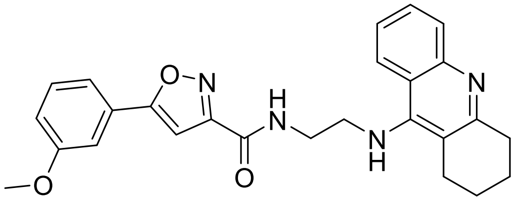 Compound 4 | training | 7.125 | 6.80468 | training | 7.09444 |
| 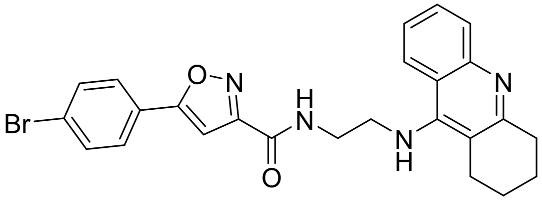 Compound 5 | training | 7.06 | 7.03326 | test | 7.16062 |
| 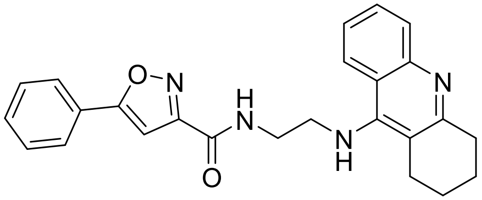 | training | 7.056 | 6.91335 | training | 7.10718 |

| | | | | | |
|---|---|---|---|---|---|
| Compound 6 | | | | | |
| 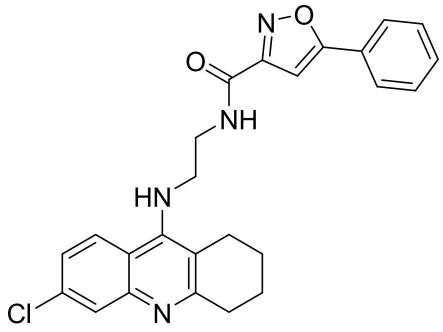<br>Compound 7 | training | 7.056 | 6.86385 | training | 7.05836 |
| 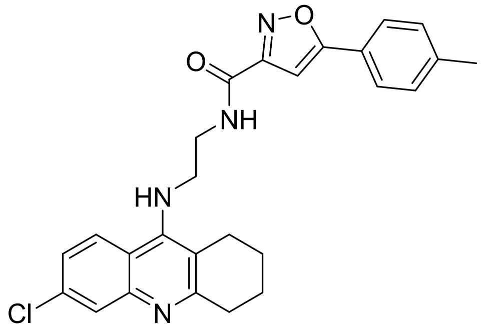<br>Compound 8 | Test | 6.983 | 6.90626 | test | 7.11863 |

| | | | | | |
|---|---|---|---|---|---|
| 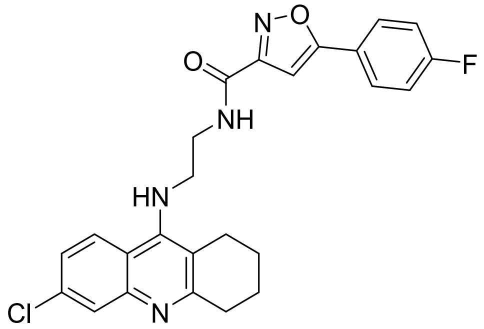 Compound 9 | training | 6.695 | 6.88183 | training | 7.08906 |
| 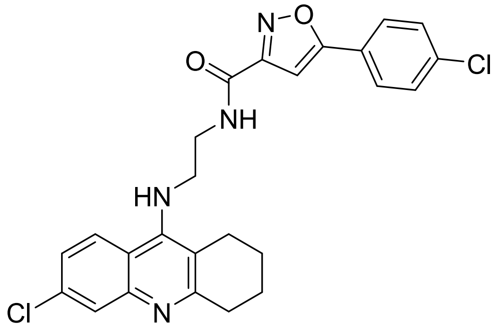 Compound 10 | Test | 6.429 | 6.96956 | training | 7.10948 |

| | | | | | |
|---|---|---|---|---|---|
| 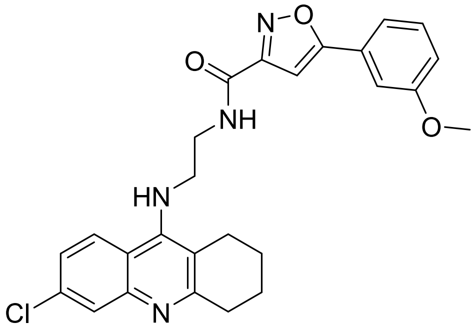 Compound 11 | training | 6.384 | 6.7491 | test | 7.04661 |
| 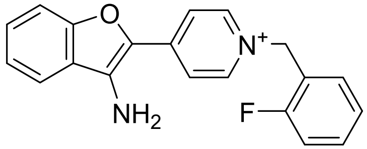 Compound 12 | training | 6.26 | 5.08415 | training | 4.96704 |
| 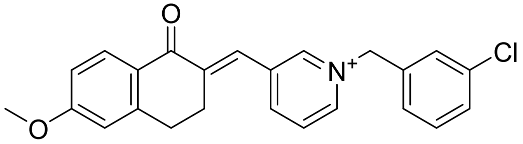 Compound 13 | training | 6.145 | 5.63036 | training | 5.37106 |
| 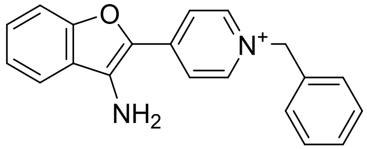 Compound 14 | training | 6.086 | 5.06577 | test | 4.9599 |

| | | | | | |
|---|---|---|---|---|---|
| 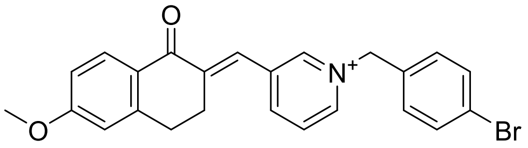<br>Compound 15 | Test | 6.054 | 5.49175 | training | 5.39839 |
| 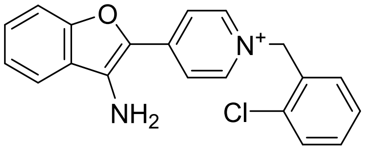<br>Compound 16 | training | 6.004 | 5.13524 | training | 4.96745 |
| 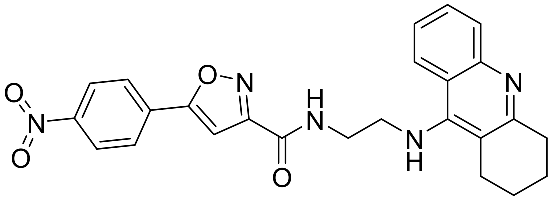<br>Compound 17 | training | 5.973 | 6.8144 | test | 7.09513 |
| 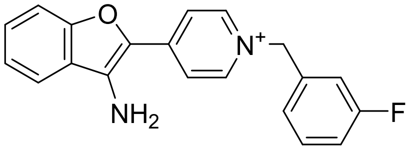<br>Compound 18 | Test | 5.539 | 5.04063 | training | 4.96209 |

| | | | | | |
|---|---|---|---|---|---|
| 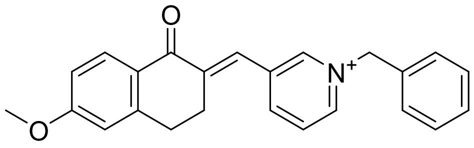 Compound 19 | training | 5.531 | 5.08863 | training | 4.9037 |
| 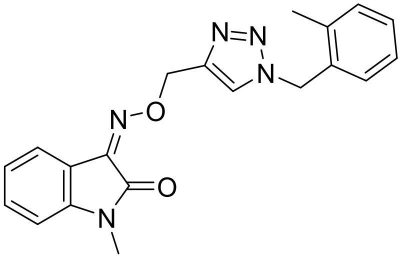 Compound 20 | training | 5.321 | 4.72085 | training | 4.66346 |
| 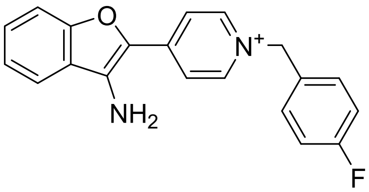 Compound 21 | Test | 5.297 | 5.02728 | training | 4.95009 |
| 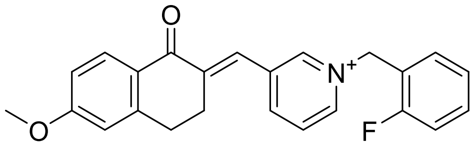 Compound 22 | training | 5.247 | 5.57774 | test | 5.35398 |

| | | | | | |
|---|---|---|---|---|---|
| 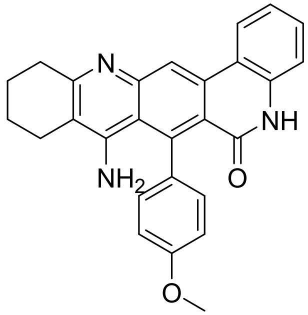 Compound 23 | Test | 5.246 | 4.16236 | training | 4.38751 |
| 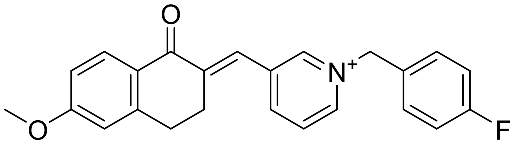 Compound 24 | training | 5.202 | 5.50657 | test | 5.39353 |
| 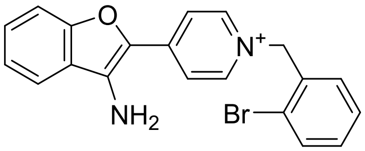 Compound 25 | training | 4.977 | 5.14092 | training | 4.96781 |
| 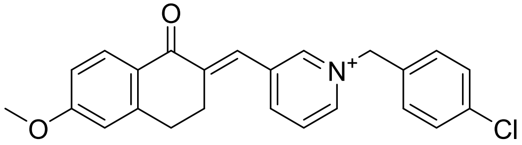 Compound 26 | training | 4.928 | 5.5032 | training | 5.39352 |

| | | | | | |
|---|---|---|---|---|---|
| 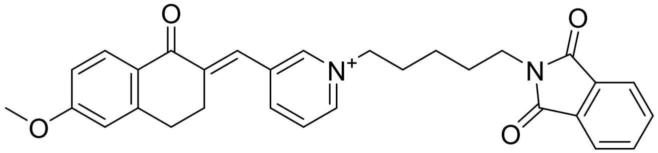 Compound 27 | training | 4.893 | 4.78543 | training | 4.96557 |
| 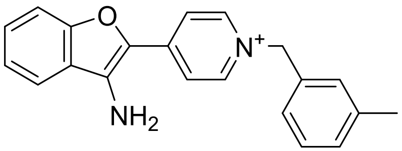 Compound 28 | Test | 4.89 | 5.09527 | test | 4.94496 |
| 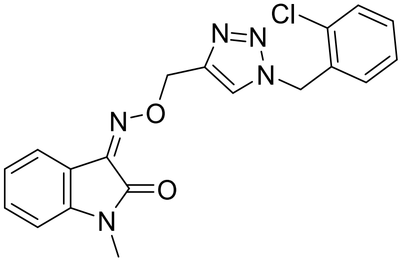 Compound 29 | training | 4.862 | 4.52889 | training | 4.56369 |
| 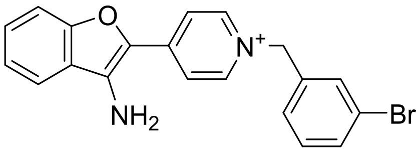 Compound 30 | training | 4.855 | 5.04828 | training | 4.94011 |

| | | | | | |
|---|---|---|---|---|---|
| 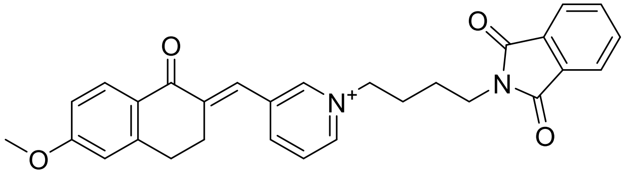 Compound 31 | Test | 4.799 | 4.75247 | test | 4.95427 |
| 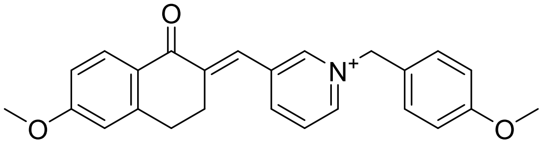 Compound 32 | training | 4.795 | 4.96831 | training | 4.90528 |
| 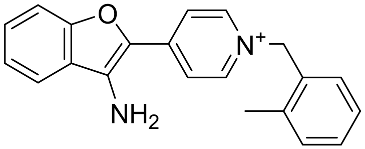 Compound 33 | Test | 4.761 | 5.07329 | training | 4.96823 |
| 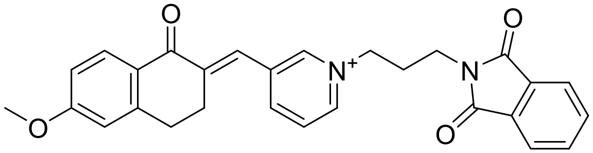 Compound 34 | training | 4.735 | 4.52077 | training | 4.9408 |

| | | | | | |
|---|---|---|---|---|---|
| 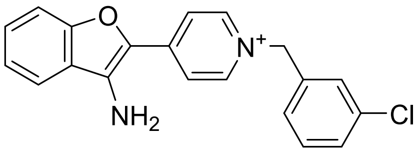 Compound 35 | training | 4.727 | 5.05447 | test | 4.94493 |
| 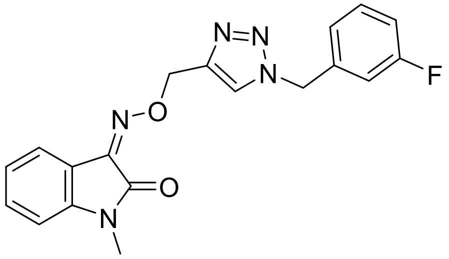 Compound 36 | training | 4.694 | 4.51215 | training | 4.55197 |
| 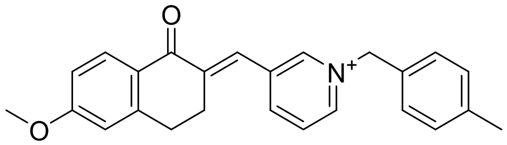 Compound 37 | Test | 4.686 | 5.06602 | test | 4.88908 |
| 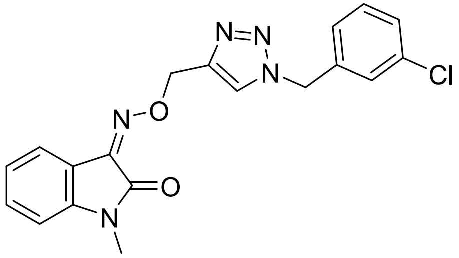 | training | 4.661 | 4.50473 | training | 4.53675 |

| | | | | | |
|---|---|---|---|---|---|
| Compound 38 | | | | | |
| 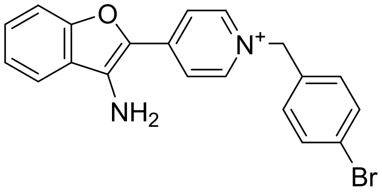<br>Compound 39 | training | 4.647 | 5.02471 | training | 4.93686 |
| 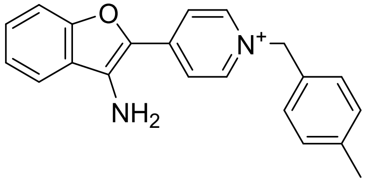<br>Compound 40 | training | 4.596 | 5.04273 | training | 4.94246 |
| 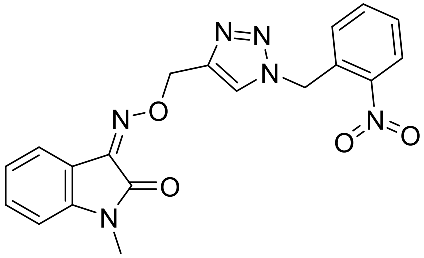<br>Compound 41 | training | 4.581 | 4.54142 | training | 4.56224 |

| | | | | | |
|---|---|---|---|---|---|
| 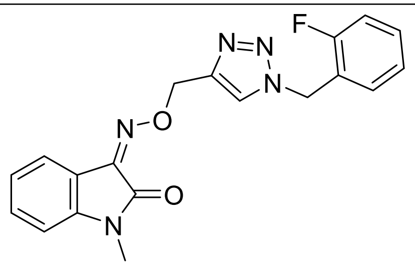 Compound 42 | Test | 4.567 | 4.52572 | training | 4.5539 |
| 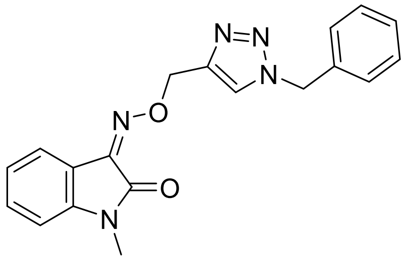 Compound 43 | Test | 4.56 | 4.61829 | test | 4.61094 |
| 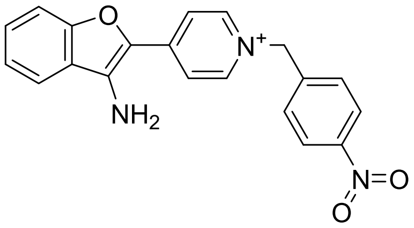 Compound 44 | training | 4.549 | 4.92555 | test | 4.95284 |

| | | | | | |
|---|---|---|---|---|---|
| 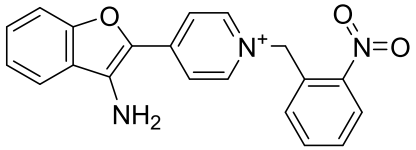 Compound 45 | training | 4.543 | 4.91151 | training | 4.8981 |
| 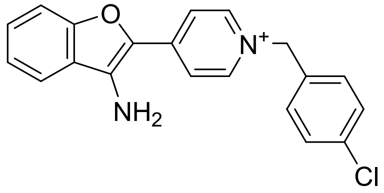 Compound 46 | training | 4.506 | 5.03493 | training | 4.9398 |
| 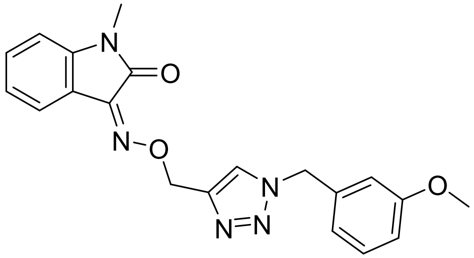 Compound 47 | training | 4.403 | 4.44188 | training | 4.54792 |

| | | | | | |
|---|---|---|---|---|---|
| 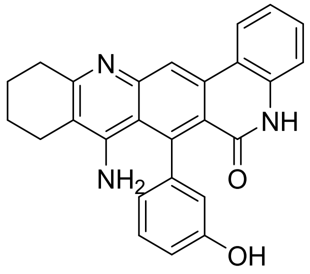<br>Compound 48 | Test | 4.378 | 4.19165 | training | 4.37168 |
| 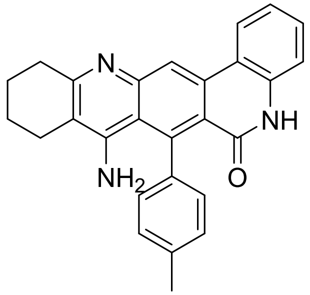<br>Compound 49 | training | 4.244 | 4.13395 | training | 4.3722 |
| 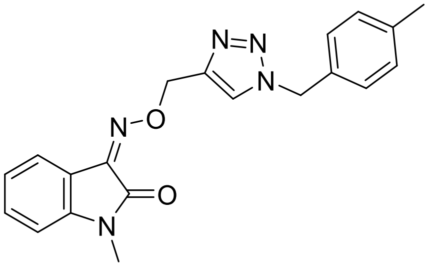<br>Compound 50 | Test | 4.208 | 4.53433 | test | 4.53704 |

| | | | | | |
|---|---|---|---|---|---|
| 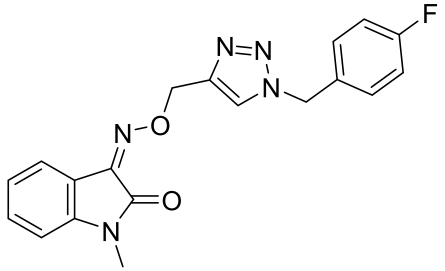 Compound 51 | training | 4 | 4.4863 | test | 4.54588 |
| 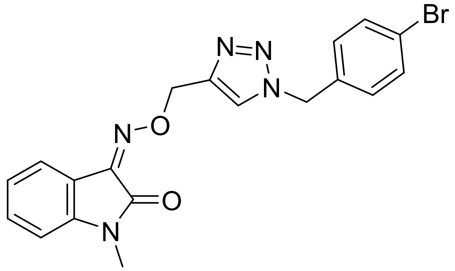 Compound 52 | Test | 4 | 4.48513 | training | 4.5214 |
| 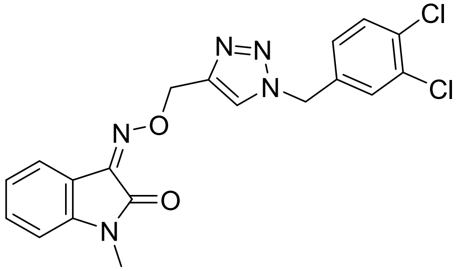 Compound 53 | training | 4 | 4.4672 | training | 4.51285 |

| | | | | | |
|---|---|---|---|---|---|
| 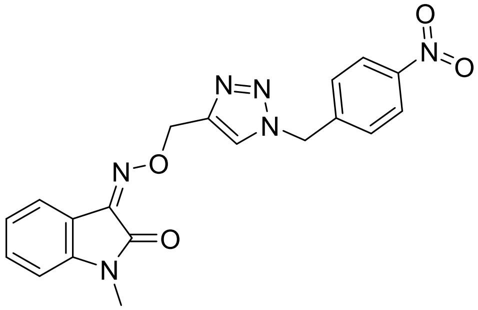 Compound 54 | training | 4 | 4.42237 | test | 4.55175 |
| 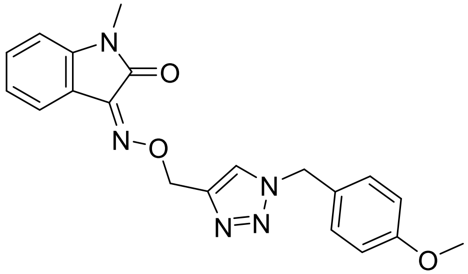 Compound 55 | training | 4 | 4.4656 | training | 4.51797 |
| 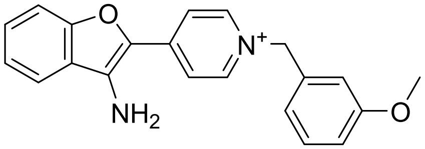 Compound 56 | Test | 4 | 4.96642 | training | 4.88115 |

| | | | | | |
|---|---|---|---|---|---|
| 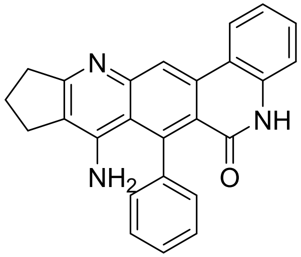<br>Compound 57 | Test | 4 | 3.79702 | training | 4.0359 |
| 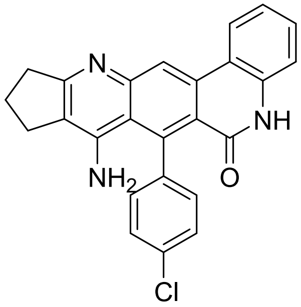<br>Compound 58 | training | 4 | 3.78282 | test | 4.04397 |
| 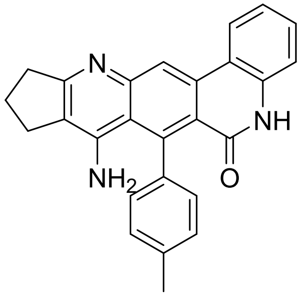<br>Compound 59 | training | 4 | 3.78324 | test | 4.04822 |

| | | | | | |
|---|---|---|---|---|---|
| 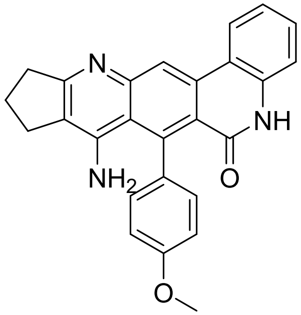 Compound 60 | Test | 4 | 3.83309 | training | 4.02512 |
| 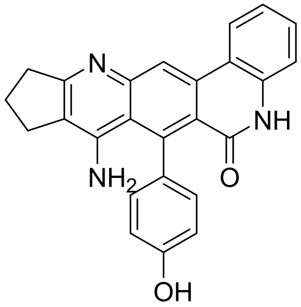 Compound 61 | training | 4 | 3.83536 | training | 4.02444 |
| 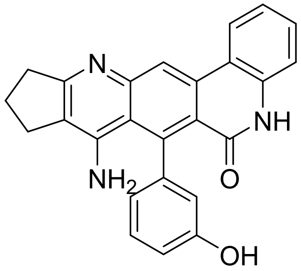 Compound 62 | training | 4 | 3.82083 | training | 4.04059 |

| | | | | | |
|---|---|---|---|---|---|
| 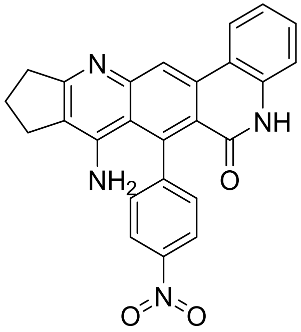 Compound 63 | training | 4 | 3.78918 | training | 4.02303 |
| 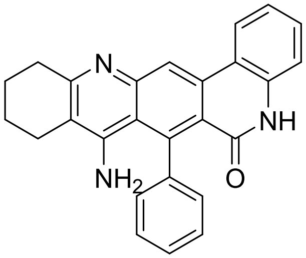 Compound 64 | training | 4 | 4.17606 | training | 4.38877 |
| 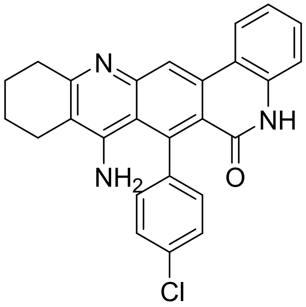 Compound 65 | training | 4 | 4.15722 | test | 4.3764 |

| | | | | | |
|---|---|---|---|---|---|
| 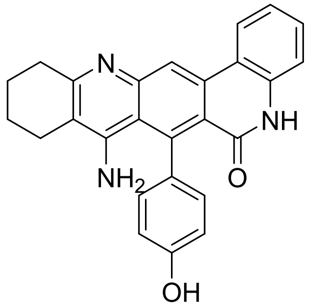 Compound 66 | Test | 4 | 4.18808 | test | 4.38807 |
| 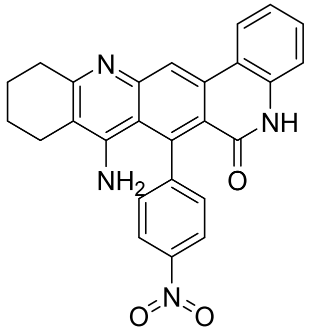 Compound 67 | training | 4 | 4.15993 | training | 4.31344 |
| 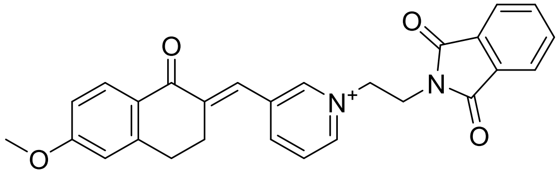 Compound 68 | training | 4 | 4.29067 | training | 4.78356 |

**Table S2**. Hit compounds with the associated smiles and docking scores.

| Title | Smiles | Docking score (XP mode) | Predicted activity 5 by Field based QSAR ($pIC_{50}$) | Estimate by 3D Pharmacophore Modeling (nM) | Fit Value for 3D pharmacophore model |
|---|---|---|---|---|---|
| Molecule5093 | Cc1ccc(C)c(N2)c1C=C(CNCc(cc1)ccc1Cl)C2=O | **-9.9396** | 4.779075529 | 484.354 | 6.32114 |
| Molecule1076 | Cc1ccc(CNCC2=Cc(c(C)ccc3C)c3NC2=O)cc1 | -9.85266 | 5.031473031 | 483.841 | 6.3216 |
| Molecule4412 | Cc1ccc(C)c(N2)c1C=C(CNCc(cc1)cc3c1OCO3)C2=O | -9.72215 | 4.703024498 | 463.151 | 6.34058 |
| Molecule1053 | Cc1ccc(C)c(N2)c1C=C(CNCc1cc(OC)ccc1)C2=O | -9.57104 | 4.888115675 | 485.046 | 6.32052 |
| Molecule3344 | Cc1ccc(C)c(N2)c1C=C(CNCc(cc1)ccc1OC)C2=O | -9.07798 | 4.918060849 | 492.702 | 6.31372 |
| Molecule1695 | Cc1ccc(C)c(N2)c1C=C(CNCc1cccc(F)c1)C2=O | -8.8853 | 4.716451827 | 483.084 | 6.32228 |
| Molecule4651 | OC1(CCN(Cc2c(C(F)(F)F)[nH]c3c2cccn3)CC1)c(cccc1)c1F | -8.84899 | 4.749806312 | 1001.64 | 6.00559 |
| Molecule3440 | Cc1ccc([C@H](c2cnccc2)NCc(cc2OC)cc3c2OCCO3)cc1 | -8.83569 | 5.065871666 | 864.521 | 6.06952 |
| Molecule4313 | CCC(N[C@H](CN(Cc1ccccc1)C1)[C@@H]1c1c(C2CC2)[nH]c2c1cccn2)=O | -8.67985 | 4.544218884 | 415.368 | 6.38787 |
| Molecule1723 | Cc1ccc(CNCC2=Cc(cc(C)cc3C)c3NC2=O)cc1 | -8.51909 | 4.86938829 | 943.076 | 6.03175 |
| Molecule4046 | CC(C)c([nH]1)c(CN(CC2)CCC2(c2ccccc2)NC(C)=O)c2c1ncc(C)c2 | -8.38236 | 5.20825585 | 970.178 | 6.01945 |
| Molecule5921 | Cc1cccc(C=C2CN(C3CCCC3)C(c3cccs3)=O)c1NC2=O | -8.26656 | 4.693281721 | 270.079 | 6.57481 |
| Molecule4676 | CCCOc(c(CN(CCC1)C[C@H]1c([nH]1)cc2c1nccc2)ccc1)c1OC | -8.15113 | 4.705824876 | 528.527 | 6.28323 |

| | | | | | |
|---|---|---|---|---|---|
| Molecule6046 | CC1(C)OCC[C@](CCNCc(cc2)cc3c2OCO3)(Cc2ccccc2)C1 | -8.13833 | 4.748465488 | 489.826 | 6.31626 |
| Molecule2163 | C[C@@H](c1nc(N2CCC(C)CC2)nc(O)c1)NCc(cc1)cc(N2C)c1N(C)C2=O | -8.12944 | 4.571676446 | 965.18 | 6.02169 |
| Molecule5712 | COc(cc1)c([C@H](CN(CCC2)[C@H]2c(cc2)cc(OC)c2OC)O)cc1F | -8.04025 | 4.752099537 | 332.473 | 6.48454 |
| Molecule4717 | CC(C)n1nc(C)c(CN(CCC2)C[C@H]2c2cc3cccnc3[nH]2)c1C | -7.8545 | 5.030468784 | 793.148 | 6.10695 |
| Molecule5470 | Cc(cc1C)cc(C=C2CNCc(cc3)cc4c3OCO4)c1NC2=O | -7.76977 | 4.700456031 | 945.072 | 6.03084 |
| Molecule4043 | Cc1cc([C@@H](CN(CC2)CCC2(c2cc(C(F)(F)F)ccc2)O)O)c(C)s1 | -7.71816 | 4.894167236 | 637.958 | 6.20151 |
| Molecule3841 | Cc1ccc([C@@H]([C@@H](CCC2)NCc3cc(OC)ccc3)N2C(C2CC2)=O)s1 | -7.68353 | 4.608620101 | 408.293 | 6.39533 |
| Molecule3943 | CN(CC1)CCN1c1cc(CCNS(c(cccc2)c2OC)(=O)=O)nc(C2CC2)n1 | -7.63122 | 4.32593379 | 332.581 | 6.4844 |
| Molecule2553 | CNc1c(CN(C)CC2)c2nc([C@@H](CCC2)CN2C(CCc2c[nH]c3c2cccc3)=O)n1 | -7.56795 | 4.583408926 | 140.624 | 6.85824 |
| Molecule2825 | Cc1nc(CC(N(CC2)CCC2(Nc2c3cccc2)NC3=O)=O)c(-c2ccccc2)s1 | -7.50992 | 4.612387659 | 217.51 | 6.66882 |
| Molecule6047 | CC(C)(C1)OCC[C@H]1[C@@H](CCNCc(cc1)cc2c1OCO2)c1ccc(C)cc1 | -7.4206 | 5.049996878 | 466.418 | 6.33752 |
| Molecule3264 | CN(C)Cc1cc([C@H](CCC2)N2C(CCCc2c[nH]c3c2cccc3)=O)nn1C | -7.3493 | 4.894776192 | 356.861 | 6.4538 |
| Molecule5679 | Cc1nn(CC=C)cc1CN(CCC1)CC[C@]1(c(cc1)ccc1F)O | -7.33668 | 5.283088576 | 329.256 | 6.48877 |
| Molecule5409 | COC(Cn1c(cccc2)c2c(CN(CC2)CCC2(c(cc2)ccc2F)O)c1)=O | -7.27092 | 5.208353134 | 914.499 | 6.04512 |
| Molecule3976 | CN(CC1)CCN1c1cc(CCNS(c(cc2)ccc2OC)(=O)=O)nc(C2CC2)n1 | -7.24062 | 4.487505846 | 609.71 | 6.22118 |
| Molecule4415 | Cc1nc(O)cc([C@H]2CN(Cc(cc(CCCC3)c3c3)c3OC)CC2)n1 | -7.16979 | 4.947756836 | 973.244 | 6.01808 |
| Molecule4407 | Cc(c(C)c1)cc2c1OC1(CCN(C[C@H](c( | -7.11615 | 4.724985896 | 108.023 | 6.97278 |

| | | | | | |
|---|---|---|---|---|---|
| | cc3)cc4c3OCCO4)O)CC1)C=C2 | | | | |
| Molecule4454 | COc(ccc([C@H](C1)c(ccc2ccccc22)c2NC1=O)c1)c1OC | -7.11384 | 4.629830094 | 395.572 | 6.40907 |
| Molecule1623 | Cc1ccc(C)c(N2)c1C=C(CNc1ccccc1)C2=O | -7.03559 | 4.737497223 | 682.246 | 6.17236 |
| Molecule4227 | Cc1ccc(C2(CCN(Cc(c(C)c3)cc(OC)c3OC)CC2)O)cc1 | -6.9741 | 4.818348073 | 362.272 | 6.44727 |
| Molecule5570 | CCOc(cc(CN(CC1)CCC1c([nH]1)cc2c1nccc2)c(C)c1)c1OC | -6.9619 | 4.613083754 | 910.706 | 6.04692 |
| Molecule5077 | Cc1ccc(C)c(N2)c1C=C(CNCc(cccc1)c1Cl)C2=O | -6.91658 | 4.769590782 | 483.04 | 6.32232 |
| Molecule2903 | CCc1noc(C)c1C(N(C[C@H]1c2cccc(F)c2)[C@H]2[C@H]1NCCCCC2)=O | -6.91614 | 5.137455039 | 588.439 | 6.2366 |
| Molecule853 | COc(cc1)c(C=C(CNCc2ccccc2)C(N2)=O)c2c1OC | -6.89497 | 4.737307427 | 507.673 | 6.30072 |
| Molecule5898 | Cc1nn2c(N)cc([C@@H]3CN(Cc(cc(cc4)OC)c4OC)CCC3)nc2c1 | -6.81438 | 4.757576926 | 96.9679 | 7.01967 |
| Molecule5415 | C[C@@H](CCN(CCC1)C[C@H]1c1nc2cc(C)nn2c(N)c1)c1ccc(C)o1 | -6.62465 | 4.726507908 | 670.846 | 6.17968 |
| Molecule4534 | Cc1noc(C)c1S(NCCc1nc(C2CC2)nc(N2CCN(C)CC2)c1)(=O)=O | -6.61864 | 4.329782793 | 501.09 | 6.30638 |
| Molecule4990 | Cc(cc1)cc(CN([C@H](CC2)C3)[C@@H]2C[C@]3(c2cccc(F)c2)O)c1OC | -6.61405 | 4.983256889 | 743.704 | 6.1349 |
| Molecule2254 | Cc1cnc(CCNS(c2cc([C@H](CC3)NC(C4CCCC4)=O)c3cc2)(=O)=O)s1 | -6.60758 | 4.609783239 | 149.195 | 6.83255 |
| Molecule4446 | Cc1c(CN(CC2)CCC2(c(cnc(OC)n2)c2OC)O)c(cccc2)c2o1 | -6.44109 | 4.425278461 | 310.776 | 6.51385 |
| Molecule5403 | COc1ccc(C2(CCCC2)NCc2c[nH]c3c2ccc(OC)c3)cc1 | -6.39607 | 4.927540192 | 375.268 | 6.43196 |
| Molecule5703 | Cc1nn2c(N)cc([C@@H]3CN(Cc(cccc4)c4OCC=C)CCC3)nc2c1 | -6.37246 | 4.611953029 | 234.88 | 6.63545 |
| Molecule4930 | Cc1c(C)sc([C@@H](Cc2cscc2)NC(c2n[nH]cc2)=O)n1 | -6.36825 | 4.54137217 | 857.82 | 6.0729 |

| | | | | | |
|---|---|---|---|---|---|
| Molecule5390 | CC(C)(C)c1csc(CCNC(c2c3nc(C4CC4)cc(C(F)F)n3nc2)=O)n1 | -6.24483 | 4.531604094 | 556.932 | 6.2605 |
| Molecule316 | Cc1nn(CC=C)cc1CN(CC1)CCC1(c1c(C)cccc1)O | -6.1694 | 4.787468789 | 612.985 | 6.21885 |
| Molecule3792 | Cc1ccc([C@@H]([C@@H](CCC2)NCc3ccccc3)N2C(C2CC2)=O)s1 | -6.01437 | 4.54331046 | 643.445 | 6.19779 |
| Molecule1436 | CCOc(ccc(CNCc1ncccc1)c1)c1OCC | -6.0086 | 4.79675048 | 372.339 | 6.43536 |
| Molecule5037 | Cc1nn(CC=C)cc1CN([C@H](CC1)C2)[C@@H]1C[C@@]2(c1cccc(F)c1)O | -5.83013 | 5.198136557 | 529.136 | 6.28273 |
| Molecule3749 | C[C@@H](Cc(cc1)cc2c1OCO2)CN(CCc1nc2cc(-c3noc(C)c3)nn22)Cc1c2O | -5.77804 | 4.925416841 | 309.642 | 6.51544 |
| Molecule4435 | Cc(c(CN([C@H](CC1)C2)[C@@H]1C[C@]2(c(cc1)ccc1OC)O)c1)cc(OC)c1OC | -5.66664 | 4.827255188 | 454.746 | 6.34853 |
| Molecule2699 | Cc(c(C)c1)cc2c1OC1(CCN(CC(c(cc3)ccc3NC(C3CC3)=O)O)CC1)C=C2 | -5.64837 | 5.103055779 | 66.6952 | 7.18221 |
| Molecule5182 | Cc1cc(CN(CCc2nc(-c3ccncc3)n3)Cc2c3O)c(C)s1 | -5.62624 | 4.663043631 | 932.383 | 6.03671 |
| Molecule223 | CCOc(ccc(CNCc1cccs1)c1)c1OC | -5.60263 | 4.874840205 | 609.791 | 6.22112 |
| Molecule5589 | COc1cc(OC)c(CN(CC2)CCc3c2c(O)nc(N2CCCC2)n3)cc1 | -5.52662 | 4.750573521 | 329.59 | 6.48833 |
| Molecule5752 | COc(ccc([C@@H]([C@H]1[C@H]2CCCC1)[C@H]2NCc1cnccc1)c1)c1OC | -5.40279 | 4.92627287 | 942.07 | 6.03222 |
| Molecule4400 | CC(C)Oc(ccc(CN([C@H](CC1)C2)[C@@H]1C[C@]2(c(cc1)ccc1F)O)c1)c1OC | -5.30529 | 5.038125981 | 816.756 | 6.09421 |
| Molecule434 | CCOc(ccc(CNCc1ccco1)c1)c1OCC | -5.2572 | 4.831791571 | 95.3676 | 7.0269 |
| Molecule2913 | CCOc(cc(CNCc1cnccc1)cc1)c1OC | -5.1791 | 4.834743702 | 91.2055 | 7.04628 |
| Molecule4635 | COc(cc1)cc(CN2CCc3nc(N4CCOCC4)nc(O)c3CC2)c1OC | -5.14847 | 5.011585408 | 342.832 | 6.47122 |
| Molecule2688 | Cc1ccc([C@@H]([C@@H](CCC2)NCc(ccc(OC)c3)c3OC)N2C(C2CC2)=O)s1 | -5.09101 | 4.945599123 | 792.002 | 6.10757 |
| Molecule4487 | CC(COc1cc(CN([C@H](CC2)C3)[C@ | -5.01601 | 4.730340726 | 704.08 | 6.15868 |

| | | | | | |
|---|---|---|---|---|---|
| | @H]2C[C@]3(c2nccn2C)O)ccc1)=C | | | | |
| Molecule82 | CCc(cc(cc1)S(Nc2cnccc2)(=O)=O)c1O CC | -4.95451 | 4.610287339 | 721.283 | 6.14819 |
| Molecule5636 | CCc(cccc1)c1NC(N[C@H]1C[C@H](C CC2)N(Cc3cccs3)[C@H]2C1)=O | -4.94066 | 4.518330864 | 507.812 | 6.3006 |
| Molecule5152 | Cc1cc(CN(CC2)Cc3c2nc(- c2ncccc2)nc3O)cc(C)c1O | -4.93352 | 5.210810478 | 723.383 | 6.14693 |
| Molecule5134 | Cc(cc1)cc(CN([C@H](CC2)C3)[C@@ H]2C[C@]3(c(cccc2)c2F)O)c1OC | -4.93034 | 5.053211128 | 835.102 | 6.08456 |
| Molecule2712 | Cc1ccc([C@@H]([C@@H](CCC2)NC c(c(OC)ccc3)c3OC)N2C(C2CC2)=O)s1 | -4.56306 | 4.788040287 | 196.749 | 6.71239 |
| Molecule4424 | Cc1nn2c(O)c(CCN(Cc3cn(CC=C)nc3C) CC3)c3nc2c1 | -4.46739 | 5.127688103 | 393.691 | 6.41114 |
| Molecule1150 | CN(c(ccc(CNCc1ccco1)c1)c1N1C)C1= O | -4.41713 | 4.763926812 | 460.353 | 6.34321 |
| Molecule3654 | CCCOc(c(C)c1)cc(C)c1S(Nc1cnccc1)(= O)=O | -3.9087 | 4.841660235 | 1000.87 | 6.00592 |
| Molecule2099 | Cc1cccc(C)c1OCC(N1Cc2c(NC)nc([C @@H]3CN(C)CCC3)nc2CC1)=O | -3.52271 | 4.269645322 | 607.706 | 6.22261 |
| Molecule3838 | Cc1ccc([C@@H]([C@@H](CCC2)NC c(cc3)ccc3OC)N2C(C2CC2)=O)s1 | -3.44294 | 4.567924802 | 99.569 | 7.00818 |
| Molecule4058 | CC(C)Oc(cc(CN1[C@H]2Cc3nc4cc(C) nn4c(O)c3C[C@@H]1CC2)cc1)c1OC | -2.83909 | 4.718920826 | 925.558 | 6.0399 |
| Molecule2091 | CC(C)n1nc(C)c(CN2C[C@H](c3c(C(N C)=O)sc4ncccc34)OCC2)c1C | -1.941 | 4.988493157 | 453.31 | 6.3499 |
| Molecule5943 | CCc(cccc1)c1NC(NC1C[C@H](CCC2) N(Cc3ccccc3)[C@@H]2C1)=O | -1.86596 | 4.878245943 | 754.657 | 6.12855 |
| Tacrine | | -6.215 | | | |

Training: 48 Prediction: 20 Variables: 740

Excluded: 0 Missing: 0 Unknown: 0

**Table S3**: Correlation matrix for QSARINS model_1_best scored.

Qi View correlation matrix

0.70 0.80 0.90

| | RDF155e | SpMax2_Bhe | MAXDP2 | ETA_Beta_ns | ETA_EtaP_B | bcutp13 |
|---|---|---|---|---|---|---|
| RDF155e | 1.0000 | | | | | |
| SpMax2_Bhe | 0.1940 | 1.0000 | | | | |
| MAXDP2 | 0.3653 | 0.1820 | 1.0000 | | | |
| ETA_Beta_ns | 0.2155 | 0.2326 | 0.3429 | 1.0000 | | |
| ETA_EtaP_B | 0.0396 | -0.1944 | 0.2473 | 0.0187 | 1.0000 | |
| bcutp13 | 0.4328 | 0.3499 | 0.7180 | 0.3063 | 0.2587 | 1.0000 |

**Table S4**: Modelling results for all QSARINS models with Var. 1 to 6 along with their statistical validations.

Qi View and select models

Sorted by R2 and by R2 QUIK rule None CI None times coeff. Only best All Best m x s All Accept Cancel

Res. std 2.5 Selected models: 1

| St. | No. | ID | Size | Variables | R2 | R2adj | R2-R2adj | LOF | Kxx | Delta K | RMSE tr | MAE tr | RSS tr | CCC tr | s | F | Q2loo |
|---|---|---|---|---|---|---|---|---|---|---|---|---|---|---|---|---|---|
| S | 1 | 198 | 6 | RDF155e SpMax2_Bhe M | 0.9083 | 0.8949 | 0.0134 | 0.1909 | 0.3471 | 0.0728 | 0.3277 | 0.2599 | 5.1553 | 0.9520 | 0.3546 | 67.7200 | 0.8729 |
| U | 2 | 197 | 6 | AATSC8i SpMax2_Bhe M | 0.9080 | 0.8945 | 0.0135 | 0.1918 | 0.4028 | 0.0725 | 0.3284 | 0.2469 | 5.1773 | 0.9518 | 0.3554 | 67.4033 | 0.8724 |
| U | 3 | 195 | 6 | SpMax2_Bhe MAXDP2 ET | 0.9075 | 0.8940 | 0.0135 | 0.1927 | 0.4310 | 0.0401 | 0.3292 | 0.2432 | 5.2025 | 0.9515 | 0.3562 | 67.0443 | 0.8723 |
| U | 4 | 199 | 6 | AATSC8i SpMax2_Bhe mi | 0.9073 | 0.8937 | 0.0136 | 0.1931 | 0.4340 | 0.0685 | 0.3296 | 0.2444 | 5.2135 | 0.9514 | 0.3566 | 66.8882 | 0.8735 |
| U | 5 | 196 | 6 | SpMax2_Bhe MAXDP2 ET | 0.9066 | 0.8930 | 0.0137 | 0.1945 | 0.3773 | 0.0509 | 0.3308 | 0.2621 | 5.2516 | 0.9510 | 0.3579 | 66.3523 | 0.8724 |
| U | 6 | 193 | 6 | AATSC8i MATS2s SpMax2 | 0.9065 | 0.8929 | 0.0137 | 0.1947 | 0.4586 | 0.0639 | 0.3309 | 0.2474 | 5.2568 | 0.9510 | 0.3581 | 66.2806 | 0.8710 |
| U | 7 | 194 | 6 | LOBMIN SpMax2_Bhe MA | 0.9060 | 0.8922 | 0.0138 | 0.1959 | 0.3908 | 0.0552 | 0.3320 | 0.2598 | 5.2899 | 0.9507 | 0.3592 | 65.8233 | 0.8711 |
| U | 8 | 192 | 6 | AATSC8i SpMax2_Bhe M | 0.9059 | 0.8921 | 0.0138 | 0.1961 | 0.4800 | 0.0580 | 0.3321 | 0.2482 | 5.2949 | 0.9506 | 0.3594 | 65.7545 | 0.8708 |
| U | 9 | 191 | 6 | LOBMIN SpMax2_Bhe MA | 0.9038 | 0.8898 | 0.0141 | 0.2003 | 0.3847 | 0.0711 | 0.3357 | 0.2492 | 5.4084 | 0.9495 | 0.3632 | 64.2316 | 0.8703 |
| U | 10 | 190 | 5 | SpMax2_Bhe VR1_Dt MA | 0.9006 | 0.8888 | 0.0118 | 0.1858 | 0.3066 | 0.1468 | 0.3412 | 0.2780 | 5.5896 | 0.9477 | 0.3648 | 76.1257 | 0.8661 |
| U | 11 | 189 | 5 | SpMax2_Bhe MAXDP2 ET | 0.9001 | 0.8882 | 0.0119 | 0.1868 | 0.4438 | 0.0728 | 0.3422 | 0.2737 | 5.6209 | 0.9474 | 0.3658 | 75.6549 | 0.8650 |
| U | 12 | 187 | 5 | SpMax2_Bhe MAXDP2 JG | 0.8982 | 0.8860 | 0.0121 | 0.1904 | 0.4433 | 0.0793 | 0.3455 | 0.2666 | 5.7283 | 0.9463 | 0.3693 | 74.0790 | 0.8634 |
| U | 13 | 188 | 5 | SpMax2_Bhe MAXDP2 ET | 0.8971 | 0.8849 | 0.0122 | 0.1923 | 0.3750 | 0.0799 | 0.3472 | 0.2691 | 5.7859 | 0.9458 | 0.3712 | 73.2573 | 0.8635 |
| U | 14 | 186 | 5 | MATS2s SpMax2_Bhe MA | 0.8965 | 0.8842 | 0.0123 | 0.1934 | 0.4068 | 0.0508 | 0.3482 | 0.2744 | 5.8189 | 0.9455 | 0.3722 | 72.7952 | 0.8632 |
| U | 15 | 183 | 5 | SpMax2_Bhe MAXDP2 ET | 0.8965 | 0.8841 | 0.0123 | 0.1936 | 0.4822 | 0.0750 | 0.3483 | 0.2673 | 5.8234 | 0.9454 | 0.3724 | 72.7321 | 0.8623 |
| U | 16 | 185 | 5 | GATS2s SpMax2_Bhe MA | 0.8963 | 0.8840 | 0.0123 | 0.1938 | 0.4429 | 0.0555 | 0.3486 | 0.2709 | 5.8316 | 0.9453 | 0.3726 | 72.6178 | 0.8631 |
| U | 17 | 184 | 5 | SpMax2_Bhe MAXDP2 ET | 0.8952 | 0.8827 | 0.0125 | 0.1959 | 0.4235 | 0.0835 | 0.3504 | 0.2714 | 5.8933 | 0.9447 | 0.3746 | 71.7700 | 0.8623 |
| U | 18 | 181 | 5 | SpMax2_Bhe MAXDP2 ET | 0.8950 | 0.8825 | 0.0125 | 0.1963 | 0.4770 | 0.0659 | 0.3507 | 0.2681 | 5.9041 | 0.9446 | 0.3749 | 71.6229 | 0.8622 |
| U | 19 | 182 | 5 | SpMax2_Bhe MAXDP2 ET | 0.8950 | 0.8825 | 0.0125 | 0.1963 | 0.4638 | 0.0792 | 0.3508 | 0.2711 | 5.9053 | 0.9446 | 0.3750 | 71.6063 | 0.8623 |
| U | 20 | 180 | 4 | SpMax2_Bhe MAXDP2 ET | 0.8833 | 0.8725 | 0.0109 | 0.1968 | 0.3841 | 0.1239 | 0.3697 | 0.2709 | 6.5615 | 0.9381 | 0.3906 | 81.4001 | 0.8522 |
| U | 21 | 179 | 4 | SpMax2_Bhe minwHBa ET | 0.8777 | 0.8663 | 0.0114 | 0.2064 | 0.4496 | 0.0920 | 0.3786 | 0.2836 | 6.8797 | 0.9349 | 0.4000 | 77.1377 | 0.8479 |
| U | 22 | 178 | 4 | MATS1s SpMax2_Bhe bcu | 0.8776 | 0.8662 | 0.0114 | 0.2066 | 0.4129 | 0.0416 | 0.3788 | 0.2997 | 6.8860 | 0.9348 | 0.4002 | 77.0577 | 0.8469 |
| U | 23 | 177 | 4 | SpMax2_Bhe MAXDP2 bc | 0.8769 | 0.8654 | 0.0115 | 0.2078 | 0.5693 | 0.0282 | 0.3798 | 0.2945 | 6.9252 | 0.9344 | 0.4013 | 76.5601 | 0.8453 |
| U | 24 | 172 | 4 | GATS1s SpMax2_Bhe ET/ | 0.8765 | 0.8650 | 0.0115 | 0.2084 | 0.4305 | 0.0967 | 0.3805 | 0.3008 | 6.9479 | 0.9342 | 0.4020 | 76.2753 | 0.8453 |
| U | 25 | 175 | 4 | SpMax2_Bhe MAXDP2 JG | 0.8736 | 0.8618 | 0.0118 | 0.2133 | 0.3338 | 0.1073 | 0.3849 | 0.2920 | 7.1100 | 0.9325 | 0.4066 | 74.2912 | 0.8393 |
| U | 26 | 176 | 4 | SpMax2_Bhe minHBint3 E | 0.8734 | 0.8617 | 0.0118 | 0.2135 | 0.4085 | 0.1247 | 0.3851 | 0.3069 | 7.1183 | 0.9324 | 0.4069 | 74.1920 | 0.8416 |
| U | 27 | 174 | 4 | SpMax2_Bhe minHBint3 J | 0.8732 | 0.8615 | 0.0118 | 0.2139 | 0.3808 | 0.0865 | 0.3854 | 0.3063 | 7.1294 | 0.9323 | 0.4072 | 74.0590 | 0.8390 |
| U | 28 | 171 | 4 | MATS3s SpMax2_Bhe ET/ | 0.8725 | 0.8606 | 0.0119 | 0.2152 | 0.3921 | 0.0555 | 0.3866 | 0.2848 | 7.1736 | 0.9319 | 0.4084 | 73.5369 | 0.8406 |
| U | 29 | 173 | 4 | SpMax2_Bhe ETA_EtaP_B | 0.8710 | 0.8590 | 0.0120 | 0.2177 | 0.4155 | 0.1154 | 0.3889 | 0.3236 | 7.2579 | 0.9310 | 0.4108 | 72.5574 | 0.8388 |
| U | 30 | 162 | 3 | SpMax2_Bhe ETA_EtaP_B | 0.8447 | 0.8342 | 0.0106 | 0.2376 | 0.2866 | 0.1761 | 0.4265 | 0.3322 | 8.7327 | 0.9158 | 0.4455 | 79.7985 | 0.8165 |
| U | 31 | 161 | 3 | SpMax2_Bhe MDEO-11 b | 0.8415 | 0.8307 | 0.0108 | 0.2426 | 0.2684 | 0.1445 | 0.4310 | 0.3455 | 8.9166 | 0.9139 | 0.4502 | 77.8503 | 0.8109 |
| U | 32 | 159 | 3 | SpMax2_Bhe JGT bcutp1 | 0.8319 | 0.8204 | 0.0115 | 0.2573 | 0.2653 | 0.0436 | 0.4439 | 0.3495 | 9.4570 | 0.9082 | 0.4636 | 72.5638 | 0.8001 |
| U | 33 | 160 | 3 | SpMax2_Bhe ETA_EtaP_B | 0.8307 | 0.8192 | 0.0115 | 0.2591 | 0.3880 | 0.0597 | 0.4454 | 0.3816 | 9.5212 | 0.9075 | 0.4652 | 71.9750 | 0.8003 |
| U | 34 | 157 | 3 | SpMax2_Bhe bcutp13 Ma | 0.8274 | 0.8156 | 0.0118 | 0.2642 | 0.3642 | 0.0775 | 0.4497 | 0.3685 | 9.7087 | 0.9055 | 0.4697 | 70.3021 | 0.7935 |
| U | 35 | 155 | 3 | SpMax2_Bhe TPSA1 bcut | 0.8269 | 0.8151 | 0.0118 | 0.2649 | 0.5427 | 0.0190 | 0.4504 | 0.3739 | 9.7361 | 0.9053 | 0.4704 | 70.0633 | 0.7923 |
| U | 36 | 158 | 3 | SpMax2_Bhe MDEN-33 b | 0.8261 | 0.8143 | 0.0119 | 0.2661 | 0.2540 | 0.1803 | 0.4514 | 0.3576 | 9.7792 | 0.9048 | 0.4714 | 69.6898 | 0.7937 |
| U | 37 | 156 | 3 | SpMax2_Bhe LipoaffinityI | 0.8243 | 0.8123 | 0.0120 | 0.2690 | 0.2904 | 0.1905 | 0.4538 | 0.3674 | 9.8843 | 0.9037 | 0.4740 | 68.7929 | 0.7923 |
| U | 38 | 154 | 3 | SpMax2_Bhe LipoaffinityI | 0.8234 | 0.8114 | 0.0120 | 0.2702 | 0.2608 | 0.1711 | 0.4548 | 0.3678 | 9.9305 | 0.9032 | 0.4751 | 68.4039 | 0.7914 |
| U | 39 | 153 | 3 | TDB3u SpMax2_Bhe bcut | 0.8229 | 0.8108 | 0.0121 | 0.2710 | 0.2725 | 0.1222 | 0.4555 | 0.3637 | 9.9605 | 0.9029 | 0.4758 | 68.1543 | 0.7909 |
| U | 40 | 152 | 2 | SpMax2_Bhe bcutp13 | 0.7973 | 0.7883 | 0.0090 | 0.2827 | 0.3877 | 0.0690 | 0.4874 | 0.3957 | 11.4032 | 0.8872 | 0.5034 | 88.4803 | 0.7675 |
| U | 41 | 150 | 2 | AATS7v GATSe4 | 0.7864 | 0.7769 | 0.0095 | 0.2979 | 0.1797 | 0.3361 | 0.5003 | 0.4180 | 12.0134 | 0.8804 | 0.5167 | 82.8432 | 0.7558 |

| No. | R2-Q2loo | RMSE cv | MAE cv | PRESS cv | CCC cv | Q2 LMO | R2 Yscr | RMSE AV Ys | Q2 Yscr | N. ext. OK | RMSE ext | MAE ext | PRESS ex | R2ex |
|---|---|---|---|---|---|---|---|---|---|---|---|---|---|---|
| 1 | 0.0355 | 0.3859 | 0.3065 | 7.1494 | 0.9338 | 0.8634 | 0.1289 | 1.0095 | -0.2023 | 3 | 0.4908 | 0.3937 | 4.8169 | 0.74 |
| 2 | 0.0355 | 0.3866 | 0.2905 | 7.1746 | 0.9337 | | | | | | 0.5050 | 0.4005 | 5.0997 | |
| 3 | 0.0352 | 0.3868 | 0.2854 | 7.1809 | 0.9335 | | | | | | 0.4694 | 0.3636 | 4.4072 | |
| 4 | 0.0338 | 0.3851 | 0.2855 | 7.1170 | 0.9339 | | | | | | 0.5408 | 0.4405 | 5.8487 | |
| 5 | 0.0342 | 0.3867 | 0.3067 | 7.1768 | 0.9334 | | | | | | 0.5018 | 0.3887 | 5.0369 | |
| 6 | 0.0356 | 0.3889 | 0.2903 | 7.2581 | 0.9328 | | | | | | 0.5656 | 0.4742 | 6.3990 | |
| 7 | 0.0348 | 0.3886 | 0.3043 | 7.2492 | 0.9327 | | | | | | 0.5122 | 0.3950 | 5.2471 | |
| 8 | 0.0351 | 0.3891 | 0.2907 | 7.2682 | 0.9324 | | | | | | 0.5477 | 0.4191 | 5.9997 | |
| 9 | 0.0335 | 0.3899 | 0.2924 | 7.2952 | 0.9321 | | | | | | 0.5018 | 0.4000 | 5.0356 | |
| 10 | 0.0345 | 0.3961 | 0.3219 | 7.5293 | 0.9294 | | | | | | 1.0218 | 0.5124 | 20.8795 | |
| 11 | 0.0351 | 0.3977 | 0.3160 | 7.5925 | 0.9290 | | | | | | 0.4669 | 0.3726 | 4.3597 | |
| 12 | 0.0348 | 0.4001 | 0.3076 | 7.6829 | 0.9285 | | | | | | 0.4920 | 0.4119 | 4.8411 | |
| 13 | 0.0336 | 0.3999 | 0.3092 | 7.6781 | 0.9283 | | | | | | 0.5013 | 0.4089 | 5.0260 | |
| 14 | 0.0333 | 0.4004 | 0.3143 | 7.6935 | 0.9282 | | | | | | 0.5709 | 0.4599 | 6.5189 | |
| 15 | 0.0342 | 0.4017 | 0.3085 | 7.7449 | 0.9277 | | | | | | 0.4919 | 0.4157 | 4.8393 | |
| 16 | 0.0332 | 0.4005 | 0.3098 | 7.6992 | 0.9280 | | | | | | 0.5273 | 0.4439 | 5.5608 | |
| 17 | 0.0329 | 0.4017 | 0.3121 | 7.7443 | 0.9276 | | | | | | 0.5101 | 0.4141 | 5.2041 | |
| 18 | 0.0328 | 0.4018 | 0.3078 | 7.7508 | 0.9275 | | | | | | 0.4962 | 0.3981 | 4.9250 | |
| 19 | 0.0327 | 0.4017 | 0.3114 | 7.7473 | 0.9274 | | | | | | 0.4949 | 0.4051 | 4.8982 | |
| 20 | 0.0312 | 0.4162 | 0.3051 | 8.3153 | 0.9215 | 0.8278 | 0.0845 | 1.0352 | -0.1424 | 2 | 0.4928 | 0.3954 | 4.8562 | 0.66 |
| 21 | 0.0298 | 0.4222 | 0.3187 | 8.5570 | 0.9191 | 0.8291 | 0.0851 | 1.0349 | -0.1422 | 1 | 0.5320 | 0.4109 | 5.6599 | 0.65 |
| 22 | 0.0306 | 0.4235 | 0.3354 | 8.6090 | 0.9186 | 0.8250 | 0.0851 | 1.0349 | -0.1454 | 2 | 0.4504 | 0.3554 | 4.0565 | 0.68 |
| 23 | 0.0316 | 0.4258 | 0.3295 | 8.7030 | 0.9180 | 0.8225 | 0.0843 | 1.0354 | -0.1451 | 0 | 0.5321 | 0.4203 | 5.6637 | 0.22 |
| 24 | 0.0312 | 0.4258 | 0.3369 | 8.7013 | 0.9176 | 0.8392 | 0.0845 | 1.0352 | -0.1422 | 1 | 0.5346 | 0.4304 | 5.7170 | 0.66 |
| 25 | 0.0343 | 0.4339 | 0.3286 | 9.0379 | 0.9141 | 0.8221 | 0.0870 | 1.0338 | -0.1396 | 1 | 0.4569 | 0.3268 | 4.1746 | 0.62 |
| 26 | 0.0319 | 0.4308 | 0.3435 | 8.9102 | 0.9155 | 0.8223 | 0.0836 | 1.0358 | -0.1442 | 2 | 0.5040 | 0.4069 | 5.0798 | 0.70 |
| 27 | 0.0342 | 0.4343 | 0.3447 | 9.0554 | 0.9141 | 0.8191 | 0.0853 | 1.0348 | -0.1421 | 3 | 0.4532 | 0.3531 | 4.1087 | 0.72 |
| 28 | 0.0318 | 0.4321 | 0.3196 | 8.9630 | 0.9149 | 0.8340 | 0.0851 | 1.0349 | -0.1427 | 1 | 0.5669 | 0.4487 | 6.4283 | 0.63 |
| 29 | 0.0322 | 0.4346 | 0.3622 | 9.0670 | 0.9139 | 0.8175 | 0.0862 | 1.0342 | -0.1403 | 1 | 0.5229 | 0.3875 | 5.4689 | 0.60 |
| 30 | 0.0283 | 0.4638 | 0.3629 | 10.3234 | 0.9005 | 0.8091 | 0.0635 | 1.0472 | -0.1167 | 1 | 0.5751 | 0.4284 | 6.6154 | 0.61 |
| 31 | 0.0306 | 0.4708 | 0.3782 | 10.6374 | 0.8981 | 0.8035 | 0.0602 | 1.0491 | -0.1222 | 1 | 0.5576 | 0.4361 | 6.2178 | 0.64 |
| 32 | 0.0318 | 0.4840 | 0.3821 | 11.2461 | 0.8907 | 0.7913 | 0.0644 | 1.0467 | -0.1159 | 1 | 0.5404 | 0.3849 | 5.8408 | 0.65 |
| 33 | 0.0304 | 0.4837 | 0.4153 | 11.2322 | 0.8911 | | | | | | 0.6254 | 0.4984 | 7.8217 | |
| 34 | 0.0338 | 0.4919 | 0.4026 | 11.6125 | 0.8870 | | | | | | 0.5088 | 0.3970 | 5.1775 | |
| 35 | 0.0346 | 0.4934 | 0.4094 | 11.6846 | 0.8869 | | | | | | 0.6592 | 0.5120 | 8.6906 | |
| 36 | 0.0324 | 0.4916 | 0.3908 | 11.6014 | 0.8874 | | | | | | 0.5101 | 0.4039 | 5.2040 | |
| 37 | 0.0319 | 0.4933 | 0.4003 | 11.6805 | 0.8862 | | | | | | 0.5762 | 0.4584 | 6.6395 | |
| 38 | 0.0320 | 0.4944 | 0.4008 | 11.7326 | 0.8865 | | | | | | 0.6069 | 0.4836 | 7.3675 | |
| 39 | 0.0320 | 0.4950 | 0.3955 | 11.7617 | 0.8853 | | | | | | 0.5110 | 0.3659 | 5.2232 | |
| 40 | 0.0298 | 0.5220 | 0.4234 | 13.0790 | 0.8707 | | | | | | 0.6109 | 0.4678 | 7.4632 | |
| 41 | 0.0306 | 0.5350 | 0.4469 | 13.7364 | 0.8632 | | | | | | 0.7950 | 0.6560 | 12.6404 | |
